# Structure of copper(II) complexes grown from ionic liquids – 1-ethyl-3-methyl­imidazolium acetate or chloride

**DOI:** 10.1107/S2056989018008538

**Published:** 2018-06-19

**Authors:** Nikita Yu. Serov, Valery G. Shtyrlin, Daut R. Islamov, Olga N. Kataeva, Dmitry B. Krivolapov

**Affiliations:** a Department of Chemistry, Kazan State University, Kremlevskaya St. 18, 420008, Kazan, Russian Federation; b Institute of Organic & Physical Chemistry, Arbuzov Str.8, 420088 Kazan, Russian Federation

**Keywords:** crystal structure, copper(II) complexes, ionic liquids, paddle-wheel

## Abstract

Crystals of four new copper(II) complexes have been grown from copper(II) acetate/chloride–1-ethyl-3-methyl­imidazolium acetate/chloride–water systems and characterized by X-ray analysis. The first complex, bis­(1-ethyl-3-methyl­imidazolium) tetra-μ-acetato-bis[chloridocuprate(II)], [Emim]_2_[Cu_2_(C_2_H_3_O_2_)_4_Cl_2_] (**1**) (Emim is 1-ethyl-3-methyl­imidazolium, C_6_H_11_N_2_), contains [Cu_2_(C_2_H_3_O_2_)_4_Cl_2_]^2−^ coordination anions with a paddle-wheel structure and ionic liquid cations. Two of the synthesized complexes are one-dimensional polymers, namely *catena*-poly[1-ethyl-3-methyl­imidazolium [[tetra-μ-acetato-dicuprate(II)]-μ-chlorido] monohydrate], {[Emim][Cu_2_(C_2_H_3_O_2_)_4_Cl]·H_2_O}_*n*_ (**2**), and *catena*-poly[1-ethyl-3-methyl­imidazolium [[tetra-μ-acetato-dicuprate(II)]-μ-acetato]], {[Emim][Cu_2_(C_2_H_3_O_2_)_5_]}_*n*_ (**3**). In these compounds, the Cu_2_(C_2_H_3_O_2_)_4_ units with a paddle-wheel structure are connected to each other through chloride (in **2**) or acetate (in **3**) anions to form parallel chains, between which cations of ionic liquid are situated. The last compound, bis­(1-ethyl-3-methyl­imidazolium) tetra-μ-acetato-bis[aquacopper(II)] tetra-μ-acetato-bis[acetatocuprate(II)] dihydrate, [Emim]_2_[Cu_2_(C_2_H_3_O_2_)_4_(H_2_O)_2_][Cu_2_(C_2_H_3_O_2_)_6_]·2H_2_O (**4**), contains two different binuclear coordination units (neutral and anionic), connected through hydrogen bonds between water mol­ecules and acetate ions.

## Abstract 2

Crystals of four new copper(II) complexes containing Cu_2_(AcO)_4_ paddle-wheel units and 1-ethyl-3-methyl­imidazolium cations have been grown from ionic liquid–water mixtures and characterized by X-ray analysis. Two of the synthesized complexes are one-dimensional coordination polymers with anionic chains and counter-ions between them.

## Chemical context   

Ionic liquids (ILs) with melting point below 373 K were discovered in 1888 (Gabriel & Weiner, 1888 ▸), but have been specific laboratory substances for a long time. However, over the past two decades ionic liquids have been of increased inter­est for researchers owing to the awareness of their unique properties, such as low dielectric permeability, low movability, wide range of liquid states, high ionic density, high ionic conductivity, good solubility for many substances, very low volatility among others (Buszewski *et al.*, 2006 ▸; Hallett & Welton, 2011 ▸). It is important that the properties of ionic liquids can be varied not only by structural design, but also by mixing with other substances, especially with water (Kohno & Ohno, 2012 ▸). The use of ILs as unique solvents for the replacement of traditional solvents and the synthesis of new substances from ionic liquids are the goals of many investigations. The application of ILs has already allowed the synthesis of new polyoxometallates, transition metal clusters, main-group element clusters and nanomaterials; the most important catalytic organic syntheses have also been performed in ionic liquids under mild conditions (Sasaki *et al.*, 2005 ▸; Ahmed & Ruck, 2011 ▸; Betz *et al.*, 2011 ▸; Jlassi *et al.*, 2014 ▸). Importantly, many oxidation reactions in organic syntheses are catalysed by copper(II) compounds, which is why the synthesis and structural investigation of copper(II) complexes grown from ILs are real scientific tasks. Of particular importance are polynuclear compounds as materials with inter­esting magnetic and electric properties.
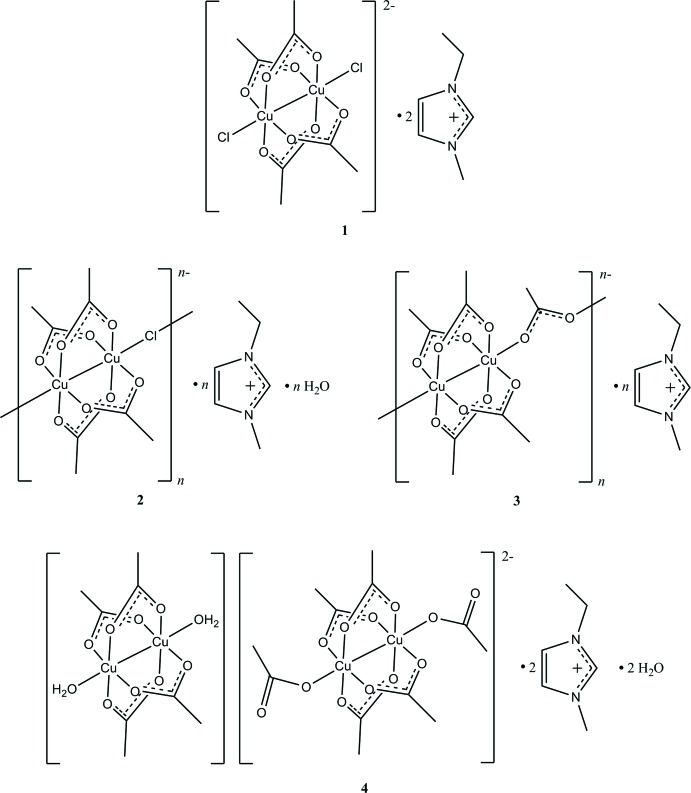


Copper(II) complexes, containing the products of ionic liquid cation C—H bond activation, have previously been isolated from the 1-ethyl-3-methyl­imidazolium acetate (EmimAcO)–copper(II) acetate [Cu(AcO)_2_]–water–air (O_2_) system in the 323–358 K temperature range (Shtyrlin *et al.*, 2014 ▸). In the present work, the new complexes **1**-**4** have been obtained from the same and similar (where the acetate ion is replaced by chloride) systems and their structures investigated by single crystal X-ray analysis.

## Structural commentary   

**Figure 1:**
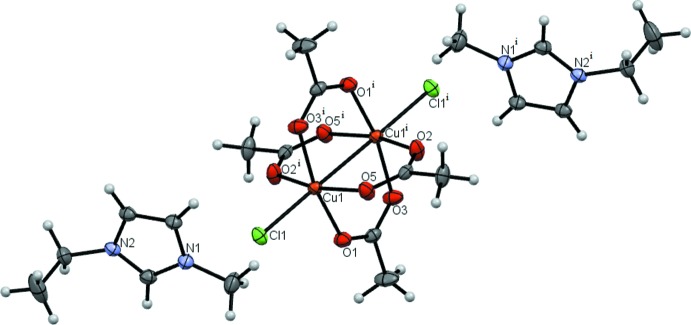
Compound **1** with displacement ellipsoids drawn at the 50% probability level. [Symmetry code: (i) −*x*, 1 − *y*, 2 − *z*.]

Compound **1** consists of two 1-ethyl-3-methyl­imidazolium cations and a binuclear complex anion [Cu_2_(AcO)_4_Cl_2_]^2−^ in which two copper(II) atoms are bonded through four bridging acetate ions. Two chloride ions are situated in the axial positions of both metal atoms, forming the axis of a paddle-wheel structure with the copper(II) ions (Fig. 1 ▸).

**Figure 2:**
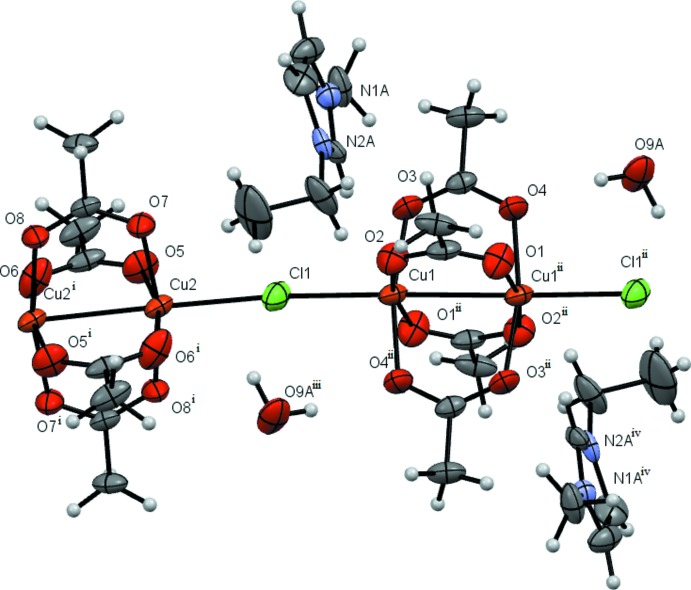
Compound **2** with displacement ellipsoids drawn at the 50% probability level. [Symmetry codes: (i) 2 − *x*, 1 − *y*, −*z*; (ii) 2 − *x*, 1 − *y*, 1 − *z*; (iii) *x*, *y*, −1 + *z*; (iv) 1 − *x*, 1 − *y*, 1 − *z*.]

Compound **2** is a polymer; in the main chain chloride ions and the two copper(II) ions, connected by four acetate ions, alternate with each other (Fig. 2 ▸). Disordered 1-ethyl-3-methyl­imidazolium cations and water mol­ecules are present in the regions between the polyanionic chains. The inter­atomic Cu⋯Cu distances in the clusters decrease (Table 1 ▸) with the transition from the binuclear compound **1** to the polymer **2**.

**Figure 3:**
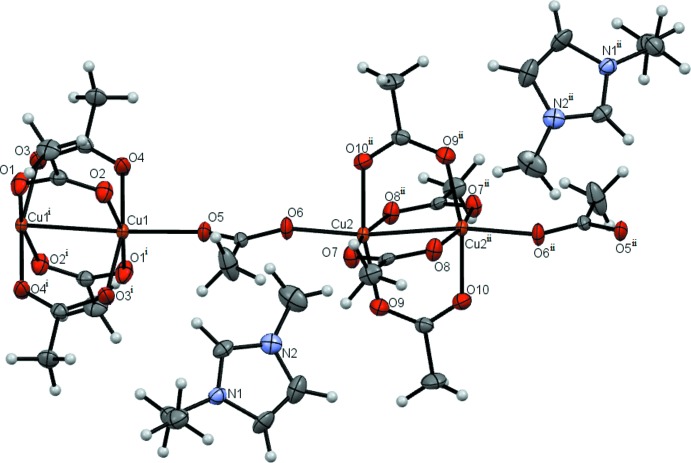
Compound **3** with displacement ellipsoids drawn at the 50% probability level. [Symmetry codes: (i) 2 − *x*, 2 − *y*, 2 − *z*; (ii) 1 − *x*, 2 − *y*, 1 − *z*.]

Compound **3** is also a polymer, but differs from **2** in the bridging ligand between clusters and the absence of water mol­ecules (Fig. 3 ▸). It is evident that the replacement of the chloride ion by acetate leads to a significant increase in the copper–copper distances between neighboring cluster units. However, the inter­atomic metal–metal distances in the clusters are practically unchanged (Table 1 ▸).

**Figure 4:**
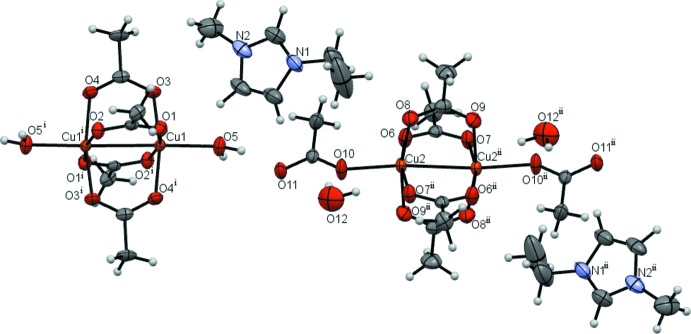
Compound **4** with displacement ellipsoids drawn at the 50% probability level. [Symmetry codes: (i) 2 − *x*, 1 − *y*, −*z*; (ii) −*x*, −*y*, 1 − *z*.]

Compound **4** has the most inter­esting structure because it contains two different clusters (Fig. 4 ▸). One of them is anionic and comprises two copper(II) ions and six acetate ions, four of which act as bridges between metal atoms. The other cluster is not charged and differs from the first by the non-bridging ligands (in this case they are water mol­ecules). Furthermore, compound **4** contains 1-ethyl-3-methyl­imidazolium ions and water mol­ecules. The metal–metal distances in the clusters in **4** are somewhat shorter than in the polymeric compounds **2** and **3** (Table 1 ▸).

## Supra­molecular features   

**Figure 5:**
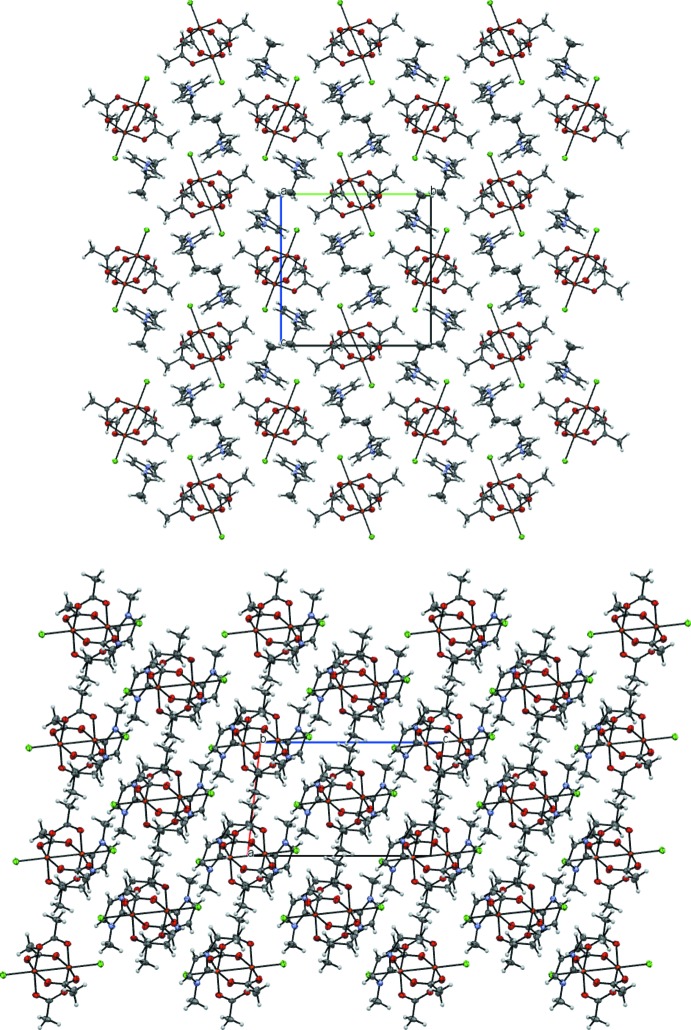
The packing of compound **1**, viewed along the *a* and *b* axes.

In the crystal of **1**, weak inter­actions are found between the [Cu_2_(AcO)_4_Cl_2_]^2−^ anion and the surrounding six 1-ethyl-3-methyl­imidazolium cations, namely C1—H1⋯O2, C2—H2⋯O5 and C3—H3⋯O3 contacts (see Table 2 ▸ for details). The last contact is relatively short and probably the strongest of them. Two different orientations of the paddle-wheels units form herringbone motif (Fig. 5 ▸).

**Figure 6:**
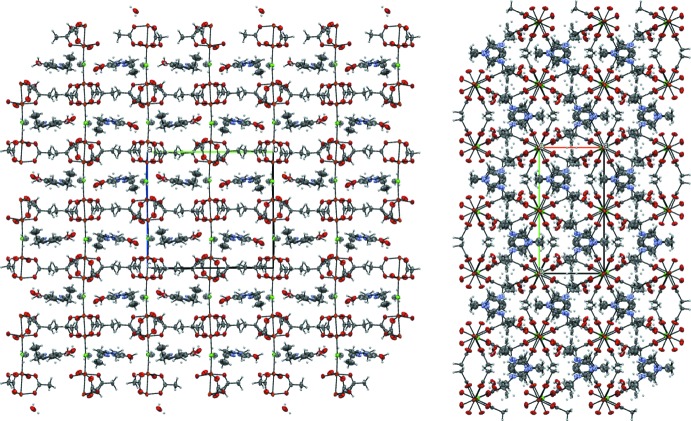
The packing of compound **2**, viewed along the *a* and *c* axes.

Polymeric chains in **2** propagate along the *c-*axis direction (Fig. 6 ▸). The water mol­ecule forms hydrogen bonds with oxygen atoms of the acetate residues of two neighbouring clusters in one chain (see Table 3 ▸). Those inter­actions decrease the Cu—Cl—Cu angle from 180° to 169.5° on the side of water mol­ecule and distort the linearity of the polymeric chains.

**Figure 7:**
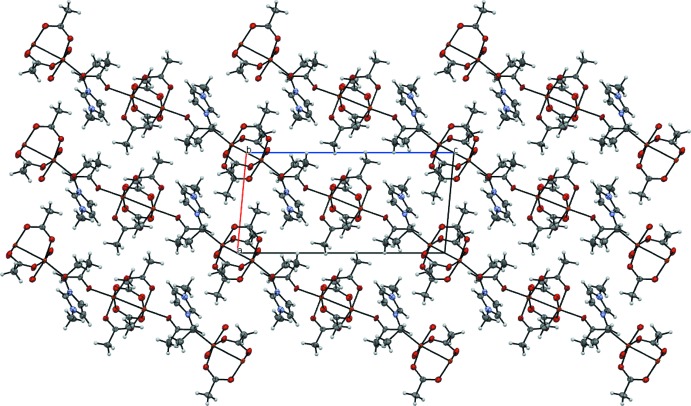
The packing of compound **3**, viewed along the *b* axis.

In **3**, the polymeric chains are not linear because neighbouring Cu_2_(AcO)_4_ fragments are connected by acetate ions (Fig. 7 ▸). The C—H⋯O inter­actions (see Table 4 ▸) between 1-ethyl-3-methyl­imidazolium cations and the anionic chains additionally stabilize the polymeric structure of **3**.

**Figure 8:**
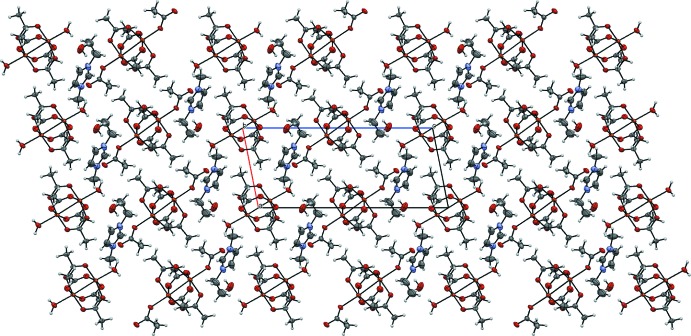
The packing of compound **4**, viewed along the *b* axis.

The crystal structure of **4** contains ordered layers (Fig. 8 ▸). Chains are formed by the alternating binuclear clusters, bonded by O—H⋯O hydrogen bonds between the coordin­ated water mol­ecules and acetate ions as ligands (O5—H5*B*⋯O11, see Table 5 ▸). The other water mol­ecule, which is not coordinated to copper(II), also plays an important role in crystal lattice formation – this water mol­ecule connects two neighbouring chains through the O5—H5⋯O12, O12—H1*O*⋯O7 and O12—H2*O*⋯O10 hydrogen bonds. The C—H⋯O inter­actions (see Table 5 ▸) between the 1-ethyl-3-methyl­imidazolium cations and acetate residues are also relevant for binding the polymeric chains.

## Database survey   

**Figure 9:**
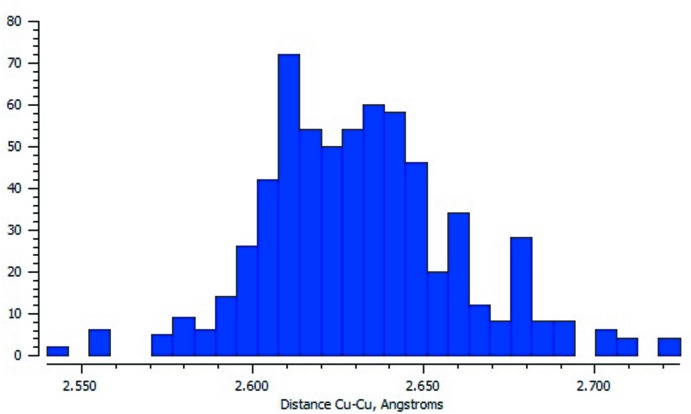
Histogram of the distribution of Cu⋯Cu distances in the Cu_2_(AcO)_4_ fragment based on a fragment search in the CSD.

A search in the Cambridge Structural Database (CSD, Version 5.58; Groom *et al.*, 2016 ▸) revealed 258 structures with the Cu_2_(AcO)_4_ fragment. In many of these structures such clusters are included several times. The distribution of Cu⋯Cu distances in such fragments is shown in Fig. 9 ▸. From a comparison of Fig. 9 ▸ and Table 1 ▸, it can be seen that the Cu⋯Cu distances in the title compounds are longer than the mean value of other structures deposited in the CSD. It should be mentioned that in **1** the Cu⋯Cu distance is very close to the maximum distance shown in Fig. 9 ▸. This long Cu⋯Cu distance can be explained by the strong inter­action between the copper(II) atoms and the chloride ions.

## Synthesis and crystallization   

**Synthesis of 1:**

A mixture of 1-ethyl-3-methyl­imidazolium acetate (0.70 g, 4.1 mmol), copper(II) chloride dihydrate (0.14 g, 0.82 mmol) and water (0.037 g, 2.05 mmol) was stirred in a closed vial at 333 K for 40 h. After several weeks, green crystals (yield 51%) were formed from the solution.

**Synthesis of 2:**

A mixture of 1-ethyl-3-methyl­imidazolium chloride (0.60 g, 4.1 mmol), copper(II) acetate hydrate (0.40 g, 2 mmol) and water (0.60 g, 33 mmol) was stirred in a closed vial at 343 K for 20 h. After several weeks, a green precipitate had formed from the solution. This precipitate consisted of crystals of compounds **1** and **2** with **1** predominant (and hence the yield of **2** was not determined).

**Synthesis of 3:**

A mixture of 1-ethyl-3-methyl­imidazolium acetate (0.70 g, 4.1 mmol) and copper(II) acetate hydrate (0.16 g, 0.80 mmol) was stirred in a closed vial at 323 K for 20 h. After several weeks, blue crystals (yield 41%) were formed from the solution.

**Synthesis of 4:**

A mixture of 1-ethyl-3-methyl­imidazolium acetate (1.0 g, 5.9 mmol), copper(II) acetate hydrate (0.078 g, 0.39 mmol) and copper(II) chloride dihydrate (0.133 g, 0.78 mmol) was stirred in a closed vial at 323 K for 30 h. After several weeks, blue crystals were formed from the solution. The yield was not determined because the precipitate additionally contained small green crystals of complex **1**. In the absence of copper(II) chloride, compound **3** was grown from the solution.

## Refinement   

Crystal data, data collection and structure refinement details are summarized in Table 6 ▸. In **2**, the Emim cations and water mol­ecules are disordered over two positions with an occupancy ratio of 0.513 (12):0.487 (12) and were refined with constraints and restraints. In **4**, the water mol­ecules refined using restraints. Water H atoms were located in difference-Fourier maps and refined using constraints with *U*_iso_(H) = 1.2*U*_eq_(O). C-bound H atoms were positioned geometrically and refined using a riding model with C—H = 0.95 (aromatic), 0.98 (methyl or 0.99 Å (methyl­ene bridges) with *U*_iso_(H) = 1.2*U*_eq_(C) or 1.5*U*_eq_(Cmeth­yl).

## Supplementary Material

Crystal structure: contains datablock(s) global, 1, 2, 3, 4. DOI: 10.1107/S2056989018008538/zp2028sup1.cif

Structure factors: contains datablock(s) 1. DOI: 10.1107/S2056989018008538/zp20281sup2.hkl

Supporting information file. DOI: 10.1107/S2056989018008538/zp20281sup6.cdx

Structure factors: contains datablock(s) 2. DOI: 10.1107/S2056989018008538/zp20282sup3.hkl

Supporting information file. DOI: 10.1107/S2056989018008538/zp20282sup7.cdx

Structure factors: contains datablock(s) 3. DOI: 10.1107/S2056989018008538/zp20283sup4.hkl

Supporting information file. DOI: 10.1107/S2056989018008538/zp20283sup8.cdx

Structure factors: contains datablock(s) 4. DOI: 10.1107/S2056989018008538/zp20284sup5.hkl

Supporting information file. DOI: 10.1107/S2056989018008538/zp20284sup9.cdx

CCDC references: 1585836, 1585835, 1585834, 1585833

Additional supporting information:  crystallographic information; 3D view; checkCIF report

## Figures and Tables

**Table 1:** Metal–metal distances (Å) in complexes **1**–**4**

Compound	Cu—Cu distance
Complex **1**	2.7173 (7)
Complex **2**	2.657 (3) and 2.669 (3)
Complex **3**	2.6571 (6) and 2.6685 (6)
Complex **4**	2.6469 (7) and 2.6592 (8)

Compounds **2**–**4** each contain two crystallographically independent clusters.

**Table 2:** Hydrogen-bond geometry (Å, °) for **1** [Chem scheme1]

*D*—H⋯*A*	*D*—H	H⋯*A*	*D*⋯*A*	*D*—H⋯*A*
C8—H8*C*⋯Cl1^i^	0.98	2.83	3.550 (3)	131
C4—H4*B*⋯Cl1	0.98	2.95	3.731 (3)	137
C4—H4*A*⋯Cl1^ii^	0.98	2.84	3.651 (3)	141
C5—H5*A*⋯Cl1^iii^	0.99	2.91	3.808 (3)	151
C2—H2⋯O5^iii^	0.95	2.57	3.295 (3)	134
C3—H3⋯O3^ii^	0.95	2.20	3.115 (3)	160
C1—H1⋯O2	0.95	2.55	3.182 (3)	124
C1—H1⋯Cl1	0.95	2.95	3.619 (3)	128

Symmetry codes: (i) 
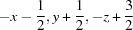
; (ii) 
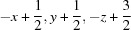
; (iii) 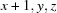.

**Table 3:** Hydrogen-bond geometry (Å, °) for **2** [Chem scheme1]

*D*—H⋯*A*	*D*—H	H⋯*A*	*D*⋯*A*	*D*—H⋯*A*
O9*B*—H2*WB*⋯O4	0.91 (2)	2.01 (2)	2.91 (2)	172 (18)
O9*A*—H1*WA*⋯O6^i^	0.90 (2)	2.3 (2)	2.94 (3)	131 (23)
O9*A*—H2*WA*⋯O4	0.90 (2)	2.19 (5)	3.08 (2)	172 (18)
C14*B*—H14*D*⋯O6^ii^	0.98	2.65	3.49 (2)	144
C12*B*—H12*B*⋯O9*B* ^iii^	0.95	2.27	3.16 (3)	155
C10*B*—H10*D*⋯Cl1^iv^	0.98	2.85	3.78 (5)	158
C9*B*—H9*B*⋯Cl1^iv^	0.95	2.84	3.67 (2)	147
C14*A*—H14*B*⋯Cl1	0.98	2.82	3.72 (3)	154
C12*A*—H12*A*⋯O9*A* ^iii^	0.95	2.19	3.13 (3)	168
C11*A*—H11*A*⋯Cl1^v^	0.95	2.88	3.77 (3)	155
C10*A*—H10*B*⋯O9*A* ^vi^	0.98	2.26	2.82 (4)	115
C10*A*—H10*A*⋯O3^iv^	0.98	2.56	3.50 (5)	161
C9*A*—H9*A*⋯O2^vii^	0.95	2.48	3.11 (3)	124
C9*A*—H9*A*⋯Cl1^iv^	0.95	2.65	3.51 (2)	151
C2—H2*C*⋯O9*B* ^vii^	0.98	2.52	3.48 (3)	165

Symmetry codes: (i) 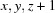; (ii) 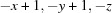; (iii) 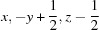; (iv) 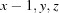; (v) 
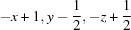
; (vi) 
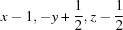
; (vii) 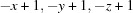.

**Table 4:** Hydrogen-bond geometry (Å, °) for **3** [Chem scheme1]

*D*—H⋯*A*	*D*—H	H⋯*A*	*D*⋯*A*	*D*—H⋯*A*
C6—H6*A*⋯O7	0.98	2.50	3.320 (4)	141
C14—H14*A*⋯O5^i^	0.99	2.47	3.329 (3)	145
C13—H13⋯O8^ii^	0.95	2.38	3.229 (4)	148
C8—H8*C*⋯O7^iii^	0.98	2.55	3.522 (4)	170
C11—H11⋯O1^iv^	0.95	2.40	3.317 (3)	162
C11—H11⋯O5	0.95	2.55	3.192 (3)	125

Symmetry codes: (i) 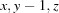; (ii) 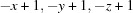; (iii) 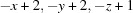; (iv) 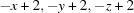.

**Table 5:** Hydrogen-bond geometry (Å, °) for **4** [Chem scheme1]

*D*—H⋯*A*	*D*—H	H⋯*A*	*D*⋯*A*	*D*—H⋯*A*
O12—H1*O*⋯O7^i^	0.91 (3)	2.21 (3)	3.034 (4)	150 (4)
O12—H2*O*⋯O10	0.87 (18)	2.09 (3)	2.910 (4)	158 (4)
O5—H5⋯O12^ii^	0.84	1.95	2.786 (5)	171
O5—H5*B*⋯O11	0.88 (3)	1.84 (3)	2.696 (3)	165 (4)
C2—H2*A*⋯O11^iii^	0.98	2.56	3.390 (4)	142
C2—H2*C*⋯O1^iii^	0.98	2.39	3.370 (4)	174
C10—H10*B*⋯O6	0.98	2.46	3.229 (4)	135
C11—H11⋯O10^iv^	0.95	2.43	3.364 (4)	166
C11—H11⋯O11^iv^	0.95	2.59	3.291 (4)	131
C12—H12⋯O1	0.95	2.31	3.234 (5)	163
C14—H14*B*⋯O7^v^	0.99	2.57	3.522 (7)	162
C16—H16*C*⋯O11^iv^	0.98	2.54	3.232 (6)	127
C16—H16*B*⋯O3	0.98	2.64	3.598 (5)	162

Symmetry codes: (i) 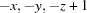; (ii) 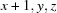; (iii) 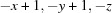; (iv) 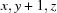; (v) 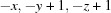.

**Table 6:** Experimental details

	**1**	**2**	**3**	**4**
Crystal data				
Chemical formula	(C_6_H_11_N_2_)_2_[Cu_2_(C_2_H_3_O_2_)_4_Cl_2_]	(C_6_H_11_N_2_)[Cu_2_(C_2_H_3_O_2_)_4_Cl]·H_2_O	(C_6_H_11_N_2_)[Cu_2_(C_2_H_3_O_2_)_5_]	(C_6_H_11_N_2_)_2_[Cu_2_(C_2_H_3_O_2_)_6_][Cu_2_(C_2_H_3_O_2_)_4_(H_2_O)_2_]·2H_2_O
*M*_r_	656.49	527.89	533.47	1139.00
Crystal system, space group	Monoclinic, *P*2_1_/*n*	Monoclinic, *P*2_1_/*c*	Triclinic, *P* 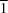	Triclinic, *P* 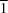
Temperature (K)	150	198	198	198
*a*, *b*, *c* (Å)	8.2264 (14), 12.956 (2), 13.173 (2)	8.438 (4), 16.315 (7), 15.131 (7)	8.0542 (9), 8.1633 (9), 16.7195 (19)	7.9526 (5), 8.0951 (5), 18.8886 (11)
α, β, γ (°)	90, 96.471 (3), 90	90, 96.53 (1), 90	98.126 (3), 94.745 (3), 92.964 (3)	79.1770 (16), 78.9500 (16), 89.9320 (15)
*V* (Å^3^)	1395.0 (4)	2069.7 (16)	1082.3 (2)	1171.46 (12)
*Z*	2	4	2	1
Radiation type	Mo *K*α	Mo *K*α	Mo *K*α	Mo *K*α
μ (mm^−1^)	1.76	2.23	2.02	1.88
Crystal size (mm)	0.30 × 0.20 × 0.20	0.11 × 0.08 × 0.07	0.30 × 0.20 × 0.20	0.30 × 0.27 × 0.22

Data collection				
Diffractometer	Bruker Kappa APEX DUO CCD	Bruker SMART APEX II CCD	Bruker Kappa APEX DUO CCD	Bruker Kappa APEX DUO CCD
Absorption correction	Multi-scan (*SADABS*; Bruker, 2015 ▸)	Multi-scan (*SADABS*; Bruker, 2015 ▸)	Multi-scan (*SADABS*; Bruker, 2015 ▸)	Multi-scan (*SADABS*; Bruker, 2015 ▸)
*T*_min_, *T*_max_	0.620, 0.719	0.795, 0.858	0.583, 0.688	0.605, 0.685
No. of measured, independent and observed [*I* > 2σ(*I*)] reflections	9428, 4275, 2956	35161, 4229, 2504	11652, 4343, 3662	20914, 4775, 3593
*R*_int_	0.039	0.105	0.025	0.037
(sin θ/λ)_max_ (Å^−1^)	0.717	0.625	0.625	0.625

Refinement				
*R*[*F* ^2^ > 2σ(*F* ^2^)], *wR*(*F* ^2^), *S*	0.041, 0.094, 1.02	0.082, 0.265, 1.08	0.029, 0.107, 0.81	0.034, 0.101, 1.42
No. of reflections	4275	4229	4343	4775
No. of parameters	167	319	278	307
No. of restraints	0	93	0	72
H-atom treatment	H-atom parameters constrained	H atoms treated by a mixture of independent and constrained refinement	H-atom parameters constrained	H atoms treated by a mixture of independent and constrained refinement
Δρ_max_, Δρ_min_ (e Å^−3^)	0.56, −0.56	1.70, −0.94	0.38, −0.46	0.40, −0.57

Computer programs: *APEX2* and *SAINT* (Bruker, 2015 ▸), *SHELXS97* (Sheldrick, 2008 ▸) and *SHELXL2014* (Sheldrick, 2015 ▸).

## supplementary crystallographic information

### Bis(1-ethyl-3-methylimidazolium) tetra-µ-acetato-bis[chloridocuprate(II)] (1) Crystal data

**Table:**

(C_6_H_11_N_2_)_2_[Cu_2_(C_2_H_3_O_2_)_4_Cl_2_]	*F*(000) = 676
*M**_r_* = 656.49	*D*_x_ = 1.563 Mg m^−^^3^
Monoclinic, *P*2_1_/*n*	Mo *K*α radiation, λ = 0.71073 Å
*a* = 8.2264 (14) Å	Cell parameters from 1837 reflections
*b* = 12.956 (2) Å	θ = 3.0–27.3°
*c* = 13.173 (2) Å	µ = 1.76 mm^−^^1^
β = 96.471 (3)°	*T* = 150 K
*V* = 1395.0 (4) Å^3^	Prism, green
*Z* = 2	0.30 × 0.20 × 0.20 mm

### Bis(1-ethyl-3-methylimidazolium) tetra-µ-acetato-bis[chloridocuprate(II)] (1) Data collection

**Table:**

Bruker Kappa APEX DUO CCD diffractometer	4275 independent reflections
Radiation source: fine-focus sealed tube	2956 reflections with *I* > 2σ(*I*)
Graphite monochromator	*R*_int_ = 0.039
φ and ω scans	θ_max_ = 30.6°, θ_min_ = 2.2°
Absorption correction: multi-scan (SADABS; Bruker, 2015)	*h* = −11→11
*T*_min_ = 0.620, *T*_max_ = 0.719	*k* = −10→18
9428 measured reflections	*l* = −18→18

### Bis(1-ethyl-3-methylimidazolium) tetra-µ-acetato-bis[chloridocuprate(II)] (1) Refinement

**Table:**

Refinement on *F*^2^	0 restraints
Least-squares matrix: full	Hydrogen site location: inferred from neighbouring sites
*R*[*F*^2^ > 2σ(*F*^2^)] = 0.041	H-atom parameters constrained
*wR*(*F*^2^) = 0.094	*w* = 1/[σ^2^(*F*_o_^2^) + (0.041*P*)^2^] where *P* = (*F*_o_^2^ + 2*F*_c_^2^)/3
*S* = 1.02	(Δ/σ)_max_ < 0.001
4275 reflections	Δρ_max_ = 0.56 e Å^−^^3^
167 parameters	Δρ_min_ = −0.56 e Å^−^^3^

### Bis(1-ethyl-3-methylimidazolium) tetra-µ-acetato-bis[chloridocuprate(II)] (1) Special details

**Table:**

Geometry. All esds (except the esd in the dihedral angle between two l.s. planes) are estimated using the full covariance matrix. The cell esds are taken into account individually in the estimation of esds in distances, angles and torsion angles; correlations between esds in cell parameters are only used when they are defined by crystal symmetry. An approximate (isotropic) treatment of cell esds is used for estimating esds involving l.s. planes.

### Bis(1-ethyl-3-methylimidazolium) tetra-µ-acetato-bis[chloridocuprate(II)] (1) Fractional atomic coordinates and isotropic or equivalent isotropic displacement parameters (Å^2^)

**Table:**

	*x*	*y*	*z*	*U*_iso_*/*U*_eq_	
Cu1	0.01642 (4)	0.45929 (2)	0.90665 (2)	0.01685 (9)	
Cl1	0.04928 (8)	0.39420 (5)	0.73731 (4)	0.02395 (15)	
C1	0.4413 (3)	0.5224 (2)	0.74431 (19)	0.0231 (6)	
H1	0.3838	0.4770	0.7842	0.028*	
C3	0.4986 (3)	0.6537 (2)	0.64963 (19)	0.0225 (5)	
H3	0.4899	0.7163	0.6119	0.027*	
C2	0.5964 (3)	0.5110 (2)	0.72166 (18)	0.0213 (5)	
H2	0.6691	0.4563	0.7432	0.026*	
C5	0.7800 (3)	0.6095 (2)	0.6145 (2)	0.0288 (6)	
H5A	0.8726	0.5765	0.6570	0.035*	
H5B	0.8027	0.6844	0.6115	0.035*	
C4	0.2199 (3)	0.6570 (2)	0.7028 (2)	0.0371 (7)	
H4A	0.2306	0.7253	0.7349	0.056*	
H4B	0.1562	0.6119	0.7431	0.056*	
H4C	0.1639	0.6637	0.6334	0.056*	
C6	0.7667 (5)	0.5654 (3)	0.5090 (3)	0.0567 (11)	
H6A	0.7434	0.4914	0.5118	0.085*	
H6B	0.8701	0.5760	0.4800	0.085*	
H6C	0.6780	0.6000	0.4661	0.085*	
N2	0.6290 (3)	0.59310 (17)	0.66186 (15)	0.0197 (4)	
N1	0.3825 (3)	0.61269 (18)	0.69846 (16)	0.0229 (5)	
C7	−0.1284 (3)	0.6607 (2)	0.91709 (19)	0.0202 (5)	
C8	−0.1941 (4)	0.7589 (2)	0.8675 (2)	0.0356 (7)	
H8A	−0.1342	0.7751	0.8093	0.053*	
H8B	−0.1806	0.8154	0.9172	0.053*	
H8C	−0.3105	0.7503	0.8436	0.053*	
O2	0.2268 (2)	0.53434 (15)	0.93042 (13)	0.0275 (4)	
O1	−0.0893 (2)	0.58958 (14)	0.86038 (13)	0.0247 (4)	
O5	−0.1994 (2)	0.39387 (15)	0.91833 (13)	0.0259 (4)	
O3	0.1164 (2)	0.34246 (14)	0.98635 (13)	0.0269 (4)	
C9	0.2752 (3)	0.5904 (2)	1.00540 (19)	0.0198 (5)	
C10	0.4370 (3)	0.6439 (2)	1.0025 (2)	0.0310 (6)	
H10A	0.5123	0.5981	0.9714	0.047*	
H10B	0.4833	0.6613	1.0722	0.047*	
H10C	0.4208	0.7073	0.9620	0.047*	

### Bis(1-ethyl-3-methylimidazolium) tetra-µ-acetato-bis[chloridocuprate(II)] (1) Atomic displacement parameters (Å^2^)

**Table:**

	*U*^11^	*U*^22^	*U*^33^	*U*^12^	*U*^13^	*U*^23^
Cu1	0.01653 (14)	0.01755 (17)	0.01630 (13)	−0.00107 (13)	0.00116 (10)	0.00019 (12)
Cl1	0.0271 (3)	0.0263 (4)	0.0192 (3)	−0.0040 (3)	0.0056 (2)	−0.0033 (2)
C1	0.0252 (13)	0.0205 (14)	0.0240 (12)	0.0004 (11)	0.0042 (10)	0.0053 (10)
C3	0.0234 (13)	0.0223 (14)	0.0214 (12)	−0.0019 (11)	0.0011 (10)	0.0031 (10)
C2	0.0263 (13)	0.0184 (13)	0.0192 (11)	0.0010 (11)	0.0019 (10)	0.0007 (10)
C5	0.0235 (13)	0.0382 (18)	0.0261 (13)	0.0003 (13)	0.0091 (10)	0.0058 (12)
C4	0.0233 (14)	0.0389 (19)	0.0506 (18)	0.0063 (14)	0.0103 (13)	0.0048 (15)
C6	0.059 (2)	0.078 (3)	0.0387 (18)	−0.010 (2)	0.0296 (17)	−0.0144 (19)
N2	0.0214 (10)	0.0201 (12)	0.0179 (9)	−0.0024 (9)	0.0029 (8)	0.0016 (8)
N1	0.0218 (11)	0.0228 (12)	0.0245 (10)	0.0015 (10)	0.0045 (8)	0.0037 (9)
C7	0.0172 (11)	0.0171 (13)	0.0252 (12)	−0.0012 (11)	−0.0022 (9)	0.0018 (10)
C8	0.0506 (19)	0.0222 (16)	0.0320 (15)	0.0109 (14)	−0.0052 (13)	0.0056 (12)
O2	0.0181 (9)	0.0361 (12)	0.0288 (9)	−0.0099 (9)	0.0053 (7)	−0.0080 (9)
O1	0.0305 (10)	0.0208 (10)	0.0225 (9)	0.0058 (9)	0.0018 (7)	0.0022 (8)
O5	0.0225 (9)	0.0322 (12)	0.0235 (9)	−0.0080 (9)	0.0041 (7)	−0.0014 (8)
O3	0.0359 (11)	0.0205 (10)	0.0230 (9)	0.0067 (9)	−0.0030 (8)	−0.0012 (8)
C9	0.0142 (11)	0.0172 (13)	0.0276 (12)	0.0010 (10)	0.0006 (9)	0.0043 (11)
C10	0.0185 (13)	0.0301 (16)	0.0458 (16)	−0.0052 (12)	0.0097 (11)	−0.0057 (14)

### Bis(1-ethyl-3-methylimidazolium) tetra-µ-acetato-bis[chloridocuprate(II)] (1) Geometric parameters (Å, º)

**Table:**

Cu1—O1	1.9642 (18)	C4—H4B	0.9800
Cu1—O3	1.9685 (18)	C4—H4C	0.9800
Cu1—O2	1.9788 (18)	C6—H6A	0.9800
Cu1—O5	1.9887 (18)	C6—H6B	0.9800
Cu1—Cl1	2.4282 (7)	C6—H6C	0.9800
Cu1—Cu1^i^	2.7173 (7)	C7—O1	1.251 (3)
C1—C2	1.351 (4)	C7—O3^i^	1.265 (3)
C1—N1	1.379 (3)	C7—C8	1.503 (4)
C1—H1	0.9500	C8—H8A	0.9800
C3—N1	1.322 (3)	C8—H8B	0.9800
C3—N2	1.324 (3)	C8—H8C	0.9800
C3—H3	0.9500	O2—C9	1.254 (3)
C2—N2	1.368 (3)	O5—C9^i^	1.258 (3)
C2—H2	0.9500	O3—C7^i^	1.265 (3)
C5—N2	1.467 (3)	C9—O5^i^	1.258 (3)
C5—C6	1.494 (4)	C9—C10	1.505 (3)
C5—H5A	0.9900	C10—H10A	0.9800
C5—H5B	0.9900	C10—H10B	0.9800
C4—N1	1.463 (3)	C10—H10C	0.9800
C4—H4A	0.9800		
			
O1—Cu1—O3	165.96 (7)	H4B—C4—H4C	109.5
O1—Cu1—O2	88.59 (8)	C5—C6—H6A	109.5
O3—Cu1—O2	89.32 (8)	C5—C6—H6B	109.5
O1—Cu1—O5	91.28 (8)	H6A—C6—H6B	109.5
O3—Cu1—O5	87.37 (8)	C5—C6—H6C	109.5
O2—Cu1—O5	165.81 (7)	H6A—C6—H6C	109.5
O1—Cu1—Cl1	96.00 (5)	H6B—C6—H6C	109.5
O3—Cu1—Cl1	98.04 (6)	C3—N2—C2	108.8 (2)
O2—Cu1—Cl1	97.48 (5)	C3—N2—C5	125.1 (2)
O5—Cu1—Cl1	96.65 (5)	C2—N2—C5	126.0 (2)
O1—Cu1—Cu1^i^	82.04 (5)	C3—N1—C1	108.5 (2)
O3—Cu1—Cu1^i^	83.92 (5)	C3—N1—C4	125.1 (2)
O2—Cu1—Cu1^i^	80.81 (5)	C1—N1—C4	126.4 (2)
O5—Cu1—Cu1^i^	85.11 (5)	O1—C7—O3^i^	125.4 (2)
Cl1—Cu1—Cu1^i^	177.41 (3)	O1—C7—C8	117.9 (2)
C2—C1—N1	106.8 (2)	O3^i^—C7—C8	116.7 (2)
C2—C1—H1	126.6	C7—C8—H8A	109.5
N1—C1—H1	126.6	C7—C8—H8B	109.5
N1—C3—N2	108.8 (2)	H8A—C8—H8B	109.5
N1—C3—H3	125.6	C7—C8—H8C	109.5
N2—C3—H3	125.6	H8A—C8—H8C	109.5
C1—C2—N2	107.1 (2)	H8B—C8—H8C	109.5
C1—C2—H2	126.5	C9—O2—Cu1	127.14 (16)
N2—C2—H2	126.5	C7—O1—Cu1	125.64 (16)
N2—C5—C6	111.3 (2)	C9^i^—O5—Cu1	121.34 (17)
N2—C5—H5A	109.4	C7^i^—O3—Cu1	122.82 (17)
C6—C5—H5A	109.4	O2—C9—O5^i^	125.5 (2)
N2—C5—H5B	109.4	O2—C9—C10	116.8 (2)
C6—C5—H5B	109.4	O5^i^—C9—C10	117.7 (2)
H5A—C5—H5B	108.0	C9—C10—H10A	109.5
N1—C4—H4A	109.5	C9—C10—H10B	109.5
N1—C4—H4B	109.5	H10A—C10—H10B	109.5
H4A—C4—H4B	109.5	C9—C10—H10C	109.5
N1—C4—H4C	109.5	H10A—C10—H10C	109.5
H4A—C4—H4C	109.5	H10B—C10—H10C	109.5

Symmetry code: (i) −*x*, −*y*+1, −*z*+2.

### Bis(1-ethyl-3-methylimidazolium) tetra-µ-acetato-bis[chloridocuprate(II)] (1) Hydrogen-bond geometry (Å, º)

**Table:**

*D*—H···*A*	*D*—H	H···*A*	*D*···*A*	*D*—H···*A*
C8—H8*C*···Cl1^ii^	0.98	2.83	3.550 (3)	131
C4—H4*B*···Cl1	0.98	2.95	3.731 (3)	137
C4—H4*A*···Cl1^iii^	0.98	2.84	3.651 (3)	141
C5—H5*A*···Cl1^iv^	0.99	2.91	3.808 (3)	151
C2—H2···O5^iv^	0.95	2.57	3.295 (3)	134
C3—H3···O3^iii^	0.95	2.20	3.115 (3)	160
C1—H1···O2	0.95	2.55	3.182 (3)	124
C1—H1···Cl1	0.95	2.95	3.619 (3)	128

Symmetry codes: (ii) −*x*−1/2, *y*+1/2, −*z*+3/2; (iii) −*x*+1/2, *y*+1/2, −*z*+3/2; (iv) *x*+1, *y*, *z*.

### catena-Poly[1-ethyl-3-methylimidazolium [[tetra-µ-acetato-dicuprate(II)]-µ-chlorido] monohydrate] (2) Crystal data

**Table:**

(C_6_H_11_N_2_)[Cu_2_(C_2_H_3_O_2_)_4_Cl]·H_2_O	*F*(000) = 1080
*M**_r_* = 527.89	*D*_x_ = 1.694 Mg m^−^^3^
Monoclinic, *P*2_1_/*c*	Mo *K*α radiation, λ = 0.71073 Å
*a* = 8.438 (4) Å	Cell parameters from 718 reflections
*b* = 16.315 (7) Å	θ = 2.4–21.6°
*c* = 15.131 (7) Å	µ = 2.23 mm^−^^1^
β = 96.53 (1)°	*T* = 198 K
*V* = 2069.7 (16) Å^3^	Prism, green
*Z* = 4	0.11 × 0.08 × 0.07 mm

### catena-Poly[1-ethyl-3-methylimidazolium [[tetra-µ-acetato-dicuprate(II)]-µ-chlorido] monohydrate] (2) Data collection

**Table:**

Bruker Smart APEX II CCD diffractometer	4229 independent reflections
Radiation source: fine-focus sealed tube	2504 reflections with *I* > 2σ(*I*)
Graphite monochromator	*R*_int_ = 0.105
φ and ω scans	θ_max_ = 26.4°, θ_min_ = 1.8°
Absorption correction: multi-scan (SADABS; Bruker, 2015)	*h* = −10→10
*T*_min_ = 0.795, *T*_max_ = 0.858	*k* = −20→20
35161 measured reflections	*l* = −16→18

### catena-Poly[1-ethyl-3-methylimidazolium [[tetra-µ-acetato-dicuprate(II)]-µ-chlorido] monohydrate] (2) Refinement

**Table:**

Refinement on *F*^2^	93 restraints
Least-squares matrix: full	Hydrogen site location: mixed
*R*[*F*^2^ > 2σ(*F*^2^)] = 0.082	H atoms treated by a mixture of independent and constrained refinement
*wR*(*F*^2^) = 0.265	*w* = 1/[σ^2^(*F*_o_^2^) + (0.1024*P*)^2^ + 19.8459*P*] where *P* = (*F*_o_^2^ + 2*F*_c_^2^)/3
*S* = 1.08	(Δ/σ)_max_ < 0.001
4229 reflections	Δρ_max_ = 1.70 e Å^−^^3^
319 parameters	Δρ_min_ = −0.94 e Å^−^^3^

### catena-Poly[1-ethyl-3-methylimidazolium [[tetra-µ-acetato-dicuprate(II)]-µ-chlorido] monohydrate] (2) Special details

**Table:**

Geometry. All esds (except the esd in the dihedral angle between two l.s. planes) are estimated using the full covariance matrix. The cell esds are taken into account individually in the estimation of esds in distances, angles and torsion angles; correlations between esds in cell parameters are only used when they are defined by crystal symmetry. An approximate (isotropic) treatment of cell esds is used for estimating esds involving l.s. planes.

### catena-Poly[1-ethyl-3-methylimidazolium [[tetra-µ-acetato-dicuprate(II)]-µ-chlorido] monohydrate] (2) Fractional atomic coordinates and isotropic or equivalent isotropic displacement parameters (Å^2^)

**Table:**

	*x*	*y*	*z*	*U*_iso_*/*U*_eq_	Occ. (<1)
C1	0.7268 (11)	0.5647 (5)	0.4896 (7)	0.035 (2)	
C2	0.5618 (11)	0.6017 (6)	0.4836 (8)	0.045 (3)	
H2A	0.5704	0.6610	0.4938	0.067*	
H2B	0.5025	0.5769	0.5289	0.067*	
H2C	0.5055	0.5913	0.4245	0.067*	
C3	0.8705 (10)	0.3595 (5)	0.5044 (7)	0.0313 (19)	
C4	0.7916 (12)	0.2770 (6)	0.5083 (8)	0.044 (3)	
H4A	0.7423	0.2618	0.4488	0.067*	
H4B	0.7096	0.2796	0.5491	0.067*	
H4C	0.8717	0.2359	0.5295	0.067*	
C5	0.7238 (11)	0.4373 (6)	−0.0335 (8)	0.039 (2)	
C6	0.5583 (11)	0.4033 (6)	−0.0541 (8)	0.047 (3)	
H6A	0.4831	0.4484	−0.0686	0.070*	
H6B	0.5286	0.3735	−0.0023	0.070*	
H6C	0.5553	0.3659	−0.1050	0.070*	
C7	1.1261 (10)	0.3598 (5)	0.0057 (6)	0.0283 (18)	
C8	1.2080 (12)	0.2769 (5)	0.0107 (7)	0.039 (2)	
H8A	1.1734	0.2453	0.0601	0.059*	
H8B	1.3239	0.2846	0.0203	0.059*	
H8C	1.1796	0.2472	−0.0452	0.059*	
N1A	0.142 (3)	0.2561 (15)	0.263 (3)	0.035 (4)	0.513 (12)
N2A	0.382 (2)	0.3105 (12)	0.2719 (13)	0.033 (3)	0.513 (12)
C9A	0.230 (2)	0.3252 (13)	0.2752 (17)	0.030 (4)	0.513 (12)
H9A	0.1871	0.3779	0.2847	0.036*	0.513 (12)
C10A	−0.029 (3)	0.248 (3)	0.264 (3)	0.050 (7)	0.513 (12)
H10A	−0.0673	0.2935	0.2989	0.075*	0.513 (12)
H10B	−0.0523	0.1960	0.2920	0.075*	0.513 (12)
H10C	−0.0817	0.2502	0.2034	0.075*	0.513 (12)
C11A	0.247 (3)	0.1942 (17)	0.246 (4)	0.048 (5)	0.513 (12)
H11A	0.2197	0.1383	0.2352	0.058*	0.513 (12)
C12A	0.391 (3)	0.2268 (14)	0.248 (5)	0.049 (5)	0.513 (12)
H12A	0.4850	0.1988	0.2357	0.059*	0.513 (12)
C13A	0.505 (3)	0.3747 (17)	0.2777 (16)	0.059 (7)	0.513 (12)
H13A	0.5941	0.3585	0.3224	0.071*	0.513 (12)
H13B	0.4592	0.4264	0.2980	0.071*	0.513 (12)
C14A	0.569 (4)	0.390 (2)	0.1885 (19)	0.105 (14)	0.513 (12)
H14A	0.6144	0.3386	0.1681	0.157*	0.513 (12)
H14B	0.6516	0.4320	0.1956	0.157*	0.513 (12)
H14C	0.4816	0.4077	0.1445	0.157*	0.513 (12)
N1B	0.193 (3)	0.2668 (17)	0.257 (4)	0.035 (4)	0.487 (12)
N2B	0.429 (2)	0.3195 (12)	0.2470 (14)	0.033 (3)	0.487 (12)
C9B	0.280 (3)	0.3363 (14)	0.2557 (18)	0.030 (4)	0.487 (12)
H9B	0.2379	0.3900	0.2605	0.036*	0.487 (12)
C10B	0.023 (3)	0.261 (3)	0.261 (4)	0.054 (9)	0.487 (12)
H10D	−0.0221	0.3163	0.2634	0.081*	0.487 (12)
H10E	0.0026	0.2302	0.3138	0.081*	0.487 (12)
H10F	−0.0270	0.2328	0.2075	0.081*	0.487 (12)
C11B	0.302 (4)	0.2041 (17)	0.251 (4)	0.048 (5)	0.487 (12)
H11B	0.2753	0.1475	0.2496	0.058*	0.487 (12)
C12B	0.448 (4)	0.2333 (15)	0.247 (6)	0.049 (5)	0.487 (12)
H12B	0.5438	0.2031	0.2453	0.059*	0.487 (12)
C13B	0.564 (3)	0.3763 (13)	0.246 (2)	0.052 (6)	0.487 (12)
H13C	0.6156	0.3674	0.1909	0.062*	0.487 (12)
H13D	0.6435	0.3656	0.2975	0.062*	0.487 (12)
C14B	0.506 (2)	0.4652 (12)	0.2484 (14)	0.042 (5)	0.487 (12)
H14D	0.3952	0.4686	0.2213	0.063*	0.487 (12)
H14E	0.5732	0.5001	0.2154	0.063*	0.487 (12)
H14F	0.5131	0.4837	0.3103	0.063*	0.487 (12)
Cl1	0.9622 (3)	0.49055 (16)	0.24814 (16)	0.0444 (6)	
Cu1	0.98839 (12)	0.49546 (7)	0.41183 (7)	0.0314 (3)	
Cu2	0.98635 (12)	0.49562 (7)	0.08689 (7)	0.0303 (3)	
O1	0.7800 (8)	0.5456 (5)	0.4194 (5)	0.0452 (17)	
O2	0.7991 (8)	0.5550 (4)	0.5671 (5)	0.0444 (17)	
O3	0.8907 (8)	0.3873 (4)	0.4302 (5)	0.0419 (16)	
O4	0.9075 (9)	0.3949 (4)	0.5780 (4)	0.0430 (17)	
O5	0.7770 (8)	0.4478 (5)	0.0462 (5)	0.0471 (18)	
O6	0.7989 (8)	0.4548 (5)	−0.0980 (5)	0.053 (2)	
O7	1.0869 (8)	0.3877 (4)	0.0774 (4)	0.0406 (16)	
O9A	0.715 (3)	0.3631 (15)	0.7359 (15)	0.056 (5)	0.513 (12)
H2WA	0.77 (2)	0.367 (12)	0.689 (10)	0.067*	0.513 (12)
H1WA	0.72 (3)	0.413 (7)	0.761 (16)	0.067*	0.513 (12)
O9B	0.691 (3)	0.4074 (14)	0.7142 (16)	0.056 (5)	0.487 (12)
H2WB	0.766 (18)	0.406 (14)	0.675 (9)	0.067*	0.487 (12)
H1WB	0.72 (4)	0.367 (19)	0.75 (2)	0.067*	0.487 (12)
O8	1.1090 (8)	0.3948 (4)	−0.0674 (4)	0.0392 (16)	

### catena-Poly[1-ethyl-3-methylimidazolium [[tetra-µ-acetato-dicuprate(II)]-µ-chlorido] monohydrate] (2) Atomic displacement parameters (Å^2^)

**Table:**

	*U*^11^	*U*^22^	*U*^33^	*U*^12^	*U*^13^	*U*^23^
C1	0.030 (4)	0.019 (4)	0.054 (6)	0.004 (3)	−0.003 (4)	−0.001 (4)
C2	0.025 (4)	0.027 (5)	0.080 (8)	−0.001 (4)	0.001 (4)	0.013 (5)
C3	0.025 (4)	0.024 (4)	0.043 (5)	0.002 (3)	−0.002 (4)	−0.005 (4)
C4	0.042 (6)	0.022 (5)	0.068 (8)	−0.004 (4)	0.003 (5)	−0.002 (5)
C5	0.025 (4)	0.024 (5)	0.067 (6)	0.005 (3)	−0.007 (4)	−0.014 (5)
C6	0.023 (4)	0.041 (6)	0.075 (8)	0.000 (4)	−0.004 (4)	−0.022 (5)
C7	0.025 (4)	0.019 (4)	0.041 (5)	−0.001 (3)	0.003 (4)	−0.007 (4)
C8	0.041 (5)	0.019 (4)	0.059 (7)	0.004 (4)	0.010 (5)	0.000 (4)
N1A	0.043 (12)	0.036 (7)	0.029 (8)	−0.007 (7)	0.008 (15)	−0.001 (7)
N2A	0.034 (8)	0.048 (5)	0.018 (10)	0.000 (5)	0.009 (5)	0.011 (6)
C9A	0.036 (9)	0.028 (6)	0.029 (11)	0.000 (6)	0.016 (8)	0.009 (7)
C10A	0.047 (13)	0.08 (2)	0.029 (14)	−0.018 (12)	0.010 (15)	0.008 (14)
C11A	0.058 (17)	0.035 (6)	0.046 (9)	0.006 (7)	−0.02 (2)	−0.008 (8)
C12A	0.046 (16)	0.049 (6)	0.049 (8)	0.023 (7)	−0.01 (3)	0.009 (9)
C13A	0.051 (14)	0.077 (13)	0.050 (15)	−0.023 (12)	0.010 (11)	0.029 (13)
C14A	0.11 (3)	0.15 (3)	0.07 (2)	−0.07 (2)	0.035 (18)	0.02 (2)
N1B	0.043 (12)	0.036 (7)	0.029 (8)	−0.007 (7)	0.008 (15)	−0.001 (7)
N2B	0.034 (8)	0.048 (5)	0.018 (10)	0.000 (5)	0.009 (5)	0.011 (6)
C9B	0.036 (9)	0.028 (6)	0.029 (11)	0.000 (6)	0.016 (8)	0.009 (7)
C10B	0.045 (14)	0.066 (19)	0.056 (19)	−0.021 (13)	0.03 (2)	0.005 (16)
C11B	0.058 (17)	0.035 (6)	0.046 (9)	0.006 (7)	−0.02 (2)	−0.008 (8)
C12B	0.046 (16)	0.049 (6)	0.049 (8)	0.023 (7)	−0.01 (3)	0.009 (9)
C13B	0.029 (11)	0.074 (11)	0.052 (19)	−0.008 (9)	0.004 (11)	0.018 (15)
C14B	0.017 (8)	0.067 (9)	0.041 (12)	−0.018 (8)	−0.002 (8)	0.005 (11)
Cl1	0.0558 (14)	0.0398 (13)	0.0342 (12)	0.0062 (11)	−0.0095 (10)	−0.0070 (11)
Cu1	0.0339 (6)	0.0200 (6)	0.0380 (7)	0.0020 (4)	−0.0066 (4)	−0.0041 (5)
Cu2	0.0289 (6)	0.0233 (6)	0.0370 (7)	0.0035 (4)	−0.0035 (4)	−0.0095 (5)
O1	0.030 (3)	0.059 (4)	0.044 (4)	0.013 (3)	−0.007 (3)	−0.006 (4)
O2	0.038 (4)	0.049 (4)	0.046 (4)	0.009 (3)	0.003 (3)	−0.003 (3)
O3	0.055 (4)	0.028 (3)	0.042 (4)	−0.009 (3)	0.003 (3)	−0.009 (3)
O4	0.062 (5)	0.028 (4)	0.038 (4)	−0.009 (3)	−0.001 (3)	−0.003 (3)
O5	0.029 (3)	0.053 (5)	0.057 (4)	−0.011 (3)	−0.006 (3)	−0.007 (4)
O6	0.031 (4)	0.074 (6)	0.053 (4)	−0.005 (4)	0.000 (3)	−0.025 (4)
O7	0.056 (4)	0.029 (3)	0.036 (4)	0.015 (3)	0.002 (3)	0.000 (3)
O9A	0.056 (8)	0.061 (12)	0.054 (11)	−0.032 (10)	0.016 (7)	−0.023 (10)
O9B	0.056 (8)	0.061 (12)	0.054 (11)	−0.032 (10)	0.016 (7)	−0.023 (10)
O8	0.057 (4)	0.025 (3)	0.038 (4)	0.013 (3)	0.015 (3)	0.001 (3)

### catena-Poly[1-ethyl-3-methylimidazolium [[tetra-µ-acetato-dicuprate(II)]-µ-chlorido] monohydrate] (2) Geometric parameters (Å, º)

**Table:**

C1—O1	1.240 (12)	C14A—H14B	0.9800
C1—O2	1.269 (12)	C14A—H14C	0.9800
C1—C2	1.511 (12)	N1B—C9B	1.348 (17)
C2—H2A	0.9800	N1B—C11B	1.382 (17)
C2—H2B	0.9800	N1B—C10B	1.45 (2)
C2—H2C	0.9800	N2B—C9B	1.309 (18)
C3—O3	1.241 (11)	N2B—C12B	1.42 (2)
C3—O4	1.261 (11)	N2B—C13B	1.47 (2)
C3—C4	1.506 (12)	C9B—H9B	0.9500
C4—H4A	0.9800	C10B—H10D	0.9800
C4—H4B	0.9800	C10B—H10E	0.9800
C4—H4C	0.9800	C10B—H10F	0.9800
C5—O5	1.250 (12)	C11B—C12B	1.33 (2)
C5—O6	1.256 (13)	C11B—H11B	0.9500
C5—C6	1.502 (12)	C12B—H12B	0.9500
C6—H6A	0.9800	C13B—C14B	1.531 (18)
C6—H6B	0.9800	C13B—H13C	0.9900
C6—H6C	0.9800	C13B—H13D	0.9900
C7—O8	1.239 (11)	C14B—H14D	0.9800
C7—O7	1.255 (11)	C14B—H14E	0.9800
C7—C8	1.518 (11)	C14B—H14F	0.9800
C8—H8A	0.9800	Cl1—Cu1	2.463 (3)
C8—H8B	0.9800	Cl1—Cu2	2.473 (3)
C8—H8C	0.9800	Cu1—O1	1.954 (7)
N1A—C9A	1.350 (17)	Cu1—O2^i^	1.966 (7)
N1A—C11A	1.383 (17)	Cu1—O3	1.981 (7)
N1A—C10A	1.45 (2)	Cu1—O4^i^	1.990 (7)
N2A—C9A	1.308 (18)	Cu1—Cu1^i^	2.657 (3)
N2A—C12A	1.42 (2)	Cu2—O5	1.965 (6)
N2A—C13A	1.47 (2)	Cu2—O7	1.967 (6)
C9A—H9A	0.9500	Cu2—O8^ii^	1.969 (6)
C10A—H10A	0.9800	Cu2—O6^ii^	1.974 (7)
C10A—H10B	0.9800	Cu2—Cu2^ii^	2.669 (3)
C10A—H10C	0.9800	O2—Cu1^i^	1.966 (7)
C11A—C12A	1.33 (2)	O4—Cu1^i^	1.990 (7)
C11A—H11A	0.9500	O6—Cu2^ii^	1.974 (7)
C12A—H12A	0.9500	O9A—H2WA	0.90 (2)
C13A—C14A	1.529 (16)	O9A—H1WA	0.90 (2)
C13A—H13A	0.9900	O9B—H2WB	0.909 (19)
C13A—H13B	0.9900	O9B—H1WB	0.90 (2)
C14A—H14A	0.9800	O8—Cu2^ii^	1.969 (6)
			
O1—C1—O2	125.3 (8)	C9B—N2B—C12B	108.6 (17)
O1—C1—C2	118.1 (9)	C9B—N2B—C13B	128.7 (18)
O2—C1—C2	116.6 (9)	C12B—N2B—C13B	122.5 (19)
C1—C2—H2A	109.5	N2B—C9B—N1B	110.6 (16)
C1—C2—H2B	109.5	N2B—C9B—H9B	124.7
H2A—C2—H2B	109.5	N1B—C9B—H9B	124.7
C1—C2—H2C	109.5	N1B—C10B—H10D	109.5
H2A—C2—H2C	109.5	N1B—C10B—H10E	109.5
H2B—C2—H2C	109.5	H10D—C10B—H10E	109.5
O3—C3—O4	125.9 (9)	N1B—C10B—H10F	109.5
O3—C3—C4	117.9 (9)	H10D—C10B—H10F	109.5
O4—C3—C4	116.2 (9)	H10E—C10B—H10F	109.5
C3—C4—H4A	109.5	C12B—C11B—N1B	111.2 (19)
C3—C4—H4B	109.5	C12B—C11B—H11B	124.4
H4A—C4—H4B	109.5	N1B—C11B—H11B	124.4
C3—C4—H4C	109.5	C11B—C12B—N2B	104.4 (19)
H4A—C4—H4C	109.5	C11B—C12B—H12B	127.8
H4B—C4—H4C	109.5	N2B—C12B—H12B	127.8
O5—C5—O6	124.2 (9)	N2B—C13B—C14B	110.3 (18)
O5—C5—C6	118.3 (10)	N2B—C13B—H13C	109.6
O6—C5—C6	117.5 (10)	C14B—C13B—H13C	109.6
C5—C6—H6A	109.5	N2B—C13B—H13D	109.6
C5—C6—H6B	109.5	C14B—C13B—H13D	109.6
H6A—C6—H6B	109.5	H13C—C13B—H13D	108.1
C5—C6—H6C	109.5	C13B—C14B—H14D	109.5
H6A—C6—H6C	109.5	C13B—C14B—H14E	109.5
H6B—C6—H6C	109.5	H14D—C14B—H14E	109.5
O8—C7—O7	126.2 (8)	C13B—C14B—H14F	109.5
O8—C7—C8	117.4 (8)	H14D—C14B—H14F	109.5
O7—C7—C8	116.3 (8)	H14E—C14B—H14F	109.5
C7—C8—H8A	109.5	Cu1—Cl1—Cu2	169.49 (13)
C7—C8—H8B	109.5	O1—Cu1—O2^i^	167.4 (3)
H8A—C8—H8B	109.5	O1—Cu1—O3	88.4 (3)
C7—C8—H8C	109.5	O2^i^—Cu1—O3	89.5 (3)
H8A—C8—H8C	109.5	O1—Cu1—O4^i^	90.7 (3)
H8B—C8—H8C	109.5	O2^i^—Cu1—O4^i^	88.7 (3)
C9A—N1A—C11A	106.5 (17)	O3—Cu1—O4^i^	167.6 (3)
C9A—N1A—C10A	127.1 (18)	O1—Cu1—Cl1	95.4 (2)
C11A—N1A—C10A	126 (2)	O2^i^—Cu1—Cl1	97.2 (2)
C9A—N2A—C12A	105.8 (17)	O3—Cu1—Cl1	96.9 (2)
C9A—N2A—C13A	123.7 (19)	O4^i^—Cu1—Cl1	95.5 (2)
C12A—N2A—C13A	129.8 (19)	O1—Cu1—Cu1^i^	83.2 (2)
N2A—C9A—N1A	111.4 (16)	O2^i^—Cu1—Cu1^i^	84.2 (2)
N2A—C9A—H9A	124.3	O3—Cu1—Cu1^i^	83.9 (2)
N1A—C9A—H9A	124.3	O4^i^—Cu1—Cu1^i^	83.7 (2)
N1A—C10A—H10A	109.5	Cl1—Cu1—Cu1^i^	178.39 (9)
N1A—C10A—H10B	109.5	O5—Cu2—O7	90.1 (3)
H10A—C10A—H10B	109.5	O5—Cu2—O8^ii^	88.6 (3)
N1A—C10A—H10C	109.5	O7—Cu2—O8^ii^	167.0 (3)
H10A—C10A—H10C	109.5	O5—Cu2—O6^ii^	166.7 (3)
H10B—C10A—H10C	109.5	O7—Cu2—O6^ii^	88.5 (3)
C12A—C11A—N1A	107.8 (19)	O8^ii^—Cu2—O6^ii^	89.8 (3)
C12A—C11A—H11A	126.1	O5—Cu2—Cl1	97.1 (2)
N1A—C11A—H11A	126.1	O7—Cu2—Cl1	97.3 (2)
C11A—C12A—N2A	108.2 (19)	O8^ii^—Cu2—Cl1	95.7 (2)
C11A—C12A—H12A	125.9	O6^ii^—Cu2—Cl1	96.2 (2)
N2A—C12A—H12A	125.9	O5—Cu2—Cu2^ii^	83.6 (2)
N2A—C13A—C14A	112 (2)	O7—Cu2—Cu2^ii^	83.7 (2)
N2A—C13A—H13A	109.2	O8^ii^—Cu2—Cu2^ii^	83.3 (2)
C14A—C13A—H13A	109.2	O6^ii^—Cu2—Cu2^ii^	83.2 (2)
N2A—C13A—H13B	109.2	Cl1—Cu2—Cu2^ii^	178.82 (9)
C14A—C13A—H13B	109.2	C1—O1—Cu1	124.9 (6)
H13A—C13A—H13B	107.9	C1—O2—Cu1^i^	122.4 (6)
C13A—C14A—H14A	109.5	C3—O3—Cu1	123.6 (6)
C13A—C14A—H14B	109.5	C3—O4—Cu1^i^	122.9 (6)
H14A—C14A—H14B	109.5	C5—O5—Cu2	124.6 (7)
C13A—C14A—H14C	109.5	C5—O6—Cu2^ii^	124.5 (6)
H14A—C14A—H14C	109.5	C7—O7—Cu2	123.0 (6)
H14B—C14A—H14C	109.5	H2WA—O9A—H1WA	105 (5)
C9B—N1B—C11B	105.1 (17)	H2WB—O9B—H1WB	105 (5)
C9B—N1B—C10B	126.4 (19)	C7—O8—Cu2^ii^	123.8 (6)
C11B—N1B—C10B	128 (2)		

Symmetry codes: (i) −*x*+2, −*y*+1, −*z*+1; (ii) −*x*+2, −*y*+1, −*z*.

### catena-Poly[1-ethyl-3-methylimidazolium [[tetra-µ-acetato-dicuprate(II)]-µ-chlorido] monohydrate] (2) Hydrogen-bond geometry (Å, º)

**Table:**

*D*—H···*A*	*D*—H	H···*A*	*D*···*A*	*D*—H···*A*
O9*B*—H2*WB*···O4	0.91 (2)	2.01 (2)	2.91 (2)	172 (18)
O9*A*—H1*WA*···O6^iii^	0.90 (2)	2.3 (2)	2.94 (3)	131 (23)
O9*A*—H2*WA*···O4	0.90 (2)	2.19 (5)	3.08 (2)	172 (18)
C14*B*—H14*D*···O6^iv^	0.98	2.65	3.49 (2)	144
C12*B*—H12*B*···O9*B*^v^	0.95	2.27	3.16 (3)	155
C10*B*—H10*D*···Cl1^vi^	0.98	2.85	3.78 (5)	158
C9*B*—H9*B*···Cl1^vi^	0.95	2.84	3.67 (2)	147
C14*A*—H14*B*···Cl1	0.98	2.82	3.72 (3)	154
C12*A*—H12*A*···O9*A*^v^	0.95	2.19	3.13 (3)	168
C11*A*—H11*A*···Cl1^vii^	0.95	2.88	3.77 (3)	155
C10*A*—H10*B*···O9*A*^viii^	0.98	2.26	2.82 (4)	115
C10*A*—H10*A*···O3^vi^	0.98	2.56	3.50 (5)	161
C9*A*—H9*A*···O2^ix^	0.95	2.48	3.11 (3)	124
C9*A*—H9*A*···Cl1^vi^	0.95	2.65	3.51 (2)	151
C2—H2*C*···O9*B*^ix^	0.98	2.52	3.48 (3)	165

Symmetry codes: (iii) *x*, *y*, *z*+1; (iv) −*x*+1, −*y*+1, −*z*; (v) *x*, −*y*+1/2, *z*−1/2; (vi) *x*−1, *y*, *z*; (vii) −*x*+1, *y*−1/2, −*z*+1/2; (viii) *x*−1, −*y*+1/2, *z*−1/2; (ix) −*x*+1, −*y*+1, −*z*+1.

### catena-Poly[1-ethyl-3-methylimidazolium [[tetra-µ-acetato-dicuprate(II)]-µ-acetato]] (3) Crystal data

**Table:**

(C_6_H_11_N_2_)[Cu_2_(C_2_H_3_O_2_)_5_]	*Z* = 2
*M**_r_* = 533.47	*F*(000) = 548
Triclinic, *P*1	*D*_x_ = 1.637 Mg m^−^^3^
*a* = 8.0542 (9) Å	Mo *K*α radiation, λ = 0.71073 Å
*b* = 8.1633 (9) Å	Cell parameters from 4553 reflections
*c* = 16.7195 (19) Å	θ = 2.5–30.5°
α = 98.126 (3)°	µ = 2.02 mm^−^^1^
β = 94.745 (3)°	*T* = 198 K
γ = 92.964 (3)°	Prism, blue
*V* = 1082.3 (2) Å^3^	0.30 × 0.20 × 0.20 mm

### catena-Poly[1-ethyl-3-methylimidazolium [[tetra-µ-acetato-dicuprate(II)]-µ-acetato]] (3) Data collection

**Table:**

Bruker Kappa APEX DUO CCD diffractometer	4343 independent reflections
Radiation source: fine-focus sealed tube	3662 reflections with *I* > 2σ(*I*)
Graphite monochromator	*R*_int_ = 0.025
φ and ω scans	θ_max_ = 26.4°, θ_min_ = 1.2°
Absorption correction: multi-scan (SADABS; Bruker, 2015)	*h* = −7→10
*T*_min_ = 0.583, *T*_max_ = 0.688	*k* = −10→10
11652 measured reflections	*l* = −20→20

### catena-Poly[1-ethyl-3-methylimidazolium [[tetra-µ-acetato-dicuprate(II)]-µ-acetato]] (3) Refinement

**Table:**

Refinement on *F*^2^	0 restraints
Least-squares matrix: full	Hydrogen site location: inferred from neighbouring sites
*R*[*F*^2^ > 2σ(*F*^2^)] = 0.029	H-atom parameters constrained
*wR*(*F*^2^) = 0.107	*w* = 1/[σ^2^(*F*_o_^2^) + (0.1*P*)^2^] where *P* = (*F*_o_^2^ + 2*F*_c_^2^)/3
*S* = 0.81	(Δ/σ)_max_ = 0.044
4343 reflections	Δρ_max_ = 0.38 e Å^−^^3^
278 parameters	Δρ_min_ = −0.46 e Å^−^^3^

### catena-Poly[1-ethyl-3-methylimidazolium [[tetra-µ-acetato-dicuprate(II)]-µ-acetato]] (3) Special details

**Table:**

Geometry. All esds (except the esd in the dihedral angle between two l.s. planes) are estimated using the full covariance matrix. The cell esds are taken into account individually in the estimation of esds in distances, angles and torsion angles; correlations between esds in cell parameters are only used when they are defined by crystal symmetry. An approximate (isotropic) treatment of cell esds is used for estimating esds involving l.s. planes.

### catena-Poly[1-ethyl-3-methylimidazolium [[tetra-µ-acetato-dicuprate(II)]-µ-acetato]] (3) Fractional atomic coordinates and isotropic or equivalent isotropic displacement parameters (Å^2^)

**Table:**

	*x*	*y*	*z*	*U*_iso_*/*U*_eq_	
Cu1	0.92345 (4)	0.99123 (3)	0.92550 (2)	0.01579 (11)	
Cu2	0.56051 (4)	0.98913 (3)	0.57530 (2)	0.01600 (11)	
O7	0.7481 (2)	0.8938 (2)	0.52154 (11)	0.0256 (4)	
O5	0.7615 (3)	0.9983 (2)	0.82103 (10)	0.0239 (4)	
O2	1.1356 (2)	1.0928 (2)	0.89944 (11)	0.0269 (4)	
O8	0.6506 (2)	0.9156 (2)	0.39488 (10)	0.0254 (4)	
O4	0.8587 (2)	1.2104 (2)	0.97397 (10)	0.0266 (4)	
O6	0.6474 (2)	1.0114 (2)	0.69902 (11)	0.0261 (4)	
O9	0.4417 (2)	0.7682 (2)	0.55935 (11)	0.0287 (5)	
O10	0.3447 (2)	0.7862 (2)	0.43204 (11)	0.0272 (4)	
O1	1.2610 (2)	1.1154 (2)	1.02491 (11)	0.0315 (5)	
O3	0.9849 (3)	1.2283 (2)	1.09893 (11)	0.0291 (5)	
N1	0.6086 (3)	0.4848 (3)	0.78982 (12)	0.0236 (5)	
N2	0.4357 (3)	0.6683 (3)	0.76515 (14)	0.0281 (5)	
C7	0.7587 (3)	0.8778 (3)	0.44641 (15)	0.0176 (5)	
C1	1.2586 (3)	1.1306 (3)	0.95090 (15)	0.0209 (5)	
C9	0.3605 (3)	0.7127 (3)	0.49278 (16)	0.0217 (5)	
C3	0.8935 (3)	1.2817 (3)	1.04474 (15)	0.0197 (5)	
C11	0.5724 (3)	0.6410 (3)	0.80908 (16)	0.0253 (6)	
H11	0.6350	0.7209	0.8484	0.030*	
C4	0.8185 (4)	1.4451 (3)	1.06738 (17)	0.0302 (6)	
H4A	0.9004	1.5360	1.0633	0.045*	
H4B	0.7874	1.4537	1.1232	0.045*	
H4C	0.7190	1.4522	1.0304	0.045*	
C5	0.7598 (3)	0.9663 (3)	0.74538 (13)	0.0191 (5)	
C8	0.9154 (3)	0.8103 (3)	0.41572 (18)	0.0305 (6)	
H8A	0.8967	0.7734	0.3571	0.046*	
H8B	0.9454	0.7162	0.4432	0.046*	
H8C	1.0064	0.8971	0.4268	0.046*	
C2	1.4171 (4)	1.1998 (4)	0.92301 (19)	0.0323 (7)	
H2A	1.5008	1.1168	0.9223	0.048*	
H2B	1.4593	1.2996	0.9602	0.048*	
H2C	1.3944	1.2280	0.8683	0.048*	
C13	0.4900 (4)	0.4082 (4)	0.73099 (17)	0.0360 (7)	
H13	0.4849	0.2961	0.7057	0.043*	
C14	0.7545 (4)	0.4061 (4)	0.82256 (17)	0.0331 (7)	
H14A	0.7178	0.2979	0.8376	0.040*	
H14B	0.8061	0.4770	0.8723	0.040*	
C15	0.8825 (4)	0.3799 (4)	0.76169 (19)	0.0360 (7)	
H15A	0.8301	0.3149	0.7113	0.054*	
H15B	0.9740	0.3201	0.7838	0.054*	
H15C	0.9267	0.4876	0.7504	0.054*	
C12	0.3825 (4)	0.5232 (4)	0.71625 (18)	0.0410 (8)	
H12	0.2863	0.5066	0.6784	0.049*	
C10	0.2743 (4)	0.5423 (3)	0.4847 (2)	0.0415 (8)	
H10A	0.3418	0.4724	0.5158	0.062*	
H10B	0.2605	0.4930	0.4274	0.062*	
H10C	0.1644	0.5506	0.5056	0.062*	
C6	0.8976 (5)	0.8706 (5)	0.71030 (18)	0.0480 (9)	
H6A	0.8610	0.8196	0.6546	0.072*	
H6B	0.9253	0.7838	0.7432	0.072*	
H6C	0.9964	0.9458	0.7102	0.072*	
C16	0.3528 (4)	0.8234 (4)	0.7685 (2)	0.0467 (9)	
H16A	0.2508	0.8140	0.7961	0.070*	
H16B	0.3242	0.8465	0.7133	0.070*	
H16C	0.4278	0.9139	0.7984	0.070*	

### catena-Poly[1-ethyl-3-methylimidazolium [[tetra-µ-acetato-dicuprate(II)]-µ-acetato]] (3) Atomic displacement parameters (Å^2^)

**Table:**

	*U*^11^	*U*^22^	*U*^33^	*U*^12^	*U*^13^	*U*^23^
Cu1	0.01738 (19)	0.01679 (18)	0.01239 (18)	0.00049 (13)	−0.00352 (12)	0.00242 (13)
Cu2	0.01685 (19)	0.01832 (18)	0.01209 (18)	0.00081 (13)	−0.00266 (13)	0.00212 (13)
O7	0.0227 (10)	0.0340 (10)	0.0206 (10)	0.0092 (8)	0.0008 (8)	0.0035 (8)
O5	0.0274 (10)	0.0272 (10)	0.0155 (9)	0.0027 (8)	−0.0073 (7)	0.0024 (7)
O2	0.0239 (11)	0.0349 (11)	0.0221 (10)	−0.0033 (8)	0.0013 (8)	0.0069 (8)
O8	0.0207 (10)	0.0351 (10)	0.0193 (9)	0.0049 (8)	−0.0002 (8)	0.0001 (8)
O4	0.0345 (12)	0.0228 (9)	0.0204 (10)	0.0101 (8)	−0.0057 (8)	−0.0020 (8)
O6	0.0273 (11)	0.0355 (11)	0.0144 (9)	0.0051 (8)	−0.0071 (8)	0.0040 (8)
O9	0.0337 (12)	0.0232 (9)	0.0291 (10)	−0.0033 (8)	−0.0020 (9)	0.0082 (8)
O10	0.0321 (11)	0.0216 (9)	0.0261 (10)	−0.0046 (8)	−0.0020 (8)	0.0028 (8)
O1	0.0248 (11)	0.0426 (12)	0.0262 (10)	−0.0107 (9)	−0.0023 (8)	0.0093 (9)
O3	0.0421 (13)	0.0211 (9)	0.0221 (10)	0.0089 (8)	−0.0072 (9)	−0.0008 (8)
N1	0.0285 (13)	0.0216 (11)	0.0193 (11)	−0.0012 (9)	0.0016 (9)	−0.0006 (9)
N2	0.0268 (13)	0.0292 (12)	0.0292 (12)	0.0016 (10)	0.0068 (10)	0.0048 (10)
C7	0.0158 (13)	0.0113 (11)	0.0236 (13)	−0.0017 (9)	0.0005 (10)	−0.0032 (9)
C1	0.0198 (14)	0.0170 (12)	0.0259 (14)	0.0004 (10)	0.0021 (11)	0.0036 (10)
C9	0.0199 (13)	0.0169 (12)	0.0287 (14)	0.0022 (10)	0.0028 (11)	0.0038 (10)
C3	0.0192 (13)	0.0184 (12)	0.0218 (13)	0.0004 (10)	0.0023 (10)	0.0043 (10)
C11	0.0234 (15)	0.0242 (13)	0.0265 (14)	−0.0063 (11)	0.0040 (11)	−0.0005 (11)
C4	0.0350 (17)	0.0220 (13)	0.0331 (15)	0.0093 (12)	0.0015 (13)	0.0000 (12)
C5	0.0247 (14)	0.0169 (11)	0.0150 (13)	−0.0028 (10)	−0.0038 (11)	0.0050 (9)
C8	0.0193 (15)	0.0312 (15)	0.0406 (17)	0.0043 (12)	0.0075 (12)	0.0005 (13)
C2	0.0230 (16)	0.0332 (15)	0.0423 (17)	−0.0027 (12)	0.0096 (13)	0.0085 (13)
C13	0.0410 (19)	0.0334 (16)	0.0273 (15)	−0.0029 (14)	−0.0010 (13)	−0.0122 (12)
C14	0.0397 (18)	0.0304 (15)	0.0307 (15)	0.0070 (13)	0.0022 (13)	0.0085 (12)
C15	0.0322 (17)	0.0310 (15)	0.0429 (18)	−0.0018 (13)	0.0015 (14)	0.0013 (13)
C12	0.0364 (19)	0.054 (2)	0.0273 (16)	−0.0020 (15)	−0.0063 (13)	−0.0040 (14)
C10	0.043 (2)	0.0216 (14)	0.057 (2)	−0.0115 (13)	−0.0064 (16)	0.0083 (14)
C6	0.053 (2)	0.073 (2)	0.0240 (15)	0.0370 (19)	0.0071 (15)	0.0124 (15)
C16	0.0346 (19)	0.048 (2)	0.065 (2)	0.0147 (15)	0.0178 (17)	0.0199 (17)

### catena-Poly[1-ethyl-3-methylimidazolium [[tetra-µ-acetato-dicuprate(II)]-µ-acetato]] (3) Geometric parameters (Å, º)

**Table:**

Cu1—O2	1.9684 (19)	C1—C2	1.505 (4)
Cu1—O4	1.9714 (18)	C9—C10	1.505 (4)
Cu1—O3^i^	1.9755 (17)	C3—C4	1.506 (3)
Cu1—O1^i^	1.9811 (19)	C11—H11	0.9500
Cu1—O5	2.1012 (17)	C4—H4A	0.9800
Cu1—Cu1^i^	2.6685 (6)	C4—H4B	0.9800
Cu2—O7	1.9607 (19)	C4—H4C	0.9800
Cu2—O9	1.9706 (18)	C5—C6	1.501 (4)
Cu2—O10^ii^	1.9742 (18)	C8—H8A	0.9800
Cu2—O8^ii^	1.9774 (18)	C8—H8B	0.9800
Cu2—O6	2.1077 (18)	C8—H8C	0.9800
Cu2—Cu2^ii^	2.6571 (6)	C2—H2A	0.9800
O7—C7	1.255 (3)	C2—H2B	0.9800
O5—C5	1.254 (3)	C2—H2C	0.9800
O2—C1	1.253 (3)	C13—C12	1.344 (4)
O8—C7	1.255 (3)	C13—H13	0.9500
O8—Cu2^ii^	1.9774 (18)	C14—C15	1.510 (4)
O4—C3	1.246 (3)	C14—H14A	0.9900
O6—C5	1.247 (3)	C14—H14B	0.9900
O9—C9	1.256 (3)	C15—H15A	0.9800
O10—C9	1.251 (3)	C15—H15B	0.9800
O10—Cu2^ii^	1.9742 (18)	C15—H15C	0.9800
O1—C1	1.260 (3)	C12—H12	0.9500
O1—Cu1^i^	1.9811 (19)	C10—H10A	0.9800
O3—C3	1.260 (3)	C10—H10B	0.9800
O3—Cu1^i^	1.9755 (17)	C10—H10C	0.9800
N1—C11	1.324 (3)	C6—H6A	0.9800
N1—C13	1.371 (4)	C6—H6B	0.9800
N1—C14	1.471 (3)	C6—H6C	0.9800
N2—C11	1.319 (3)	C16—H16A	0.9800
N2—C12	1.368 (4)	C16—H16B	0.9800
N2—C16	1.458 (4)	C16—H16C	0.9800
C7—C8	1.503 (4)		
			
O2—Cu1—O4	90.22 (9)	N2—C11—H11	125.3
O2—Cu1—O3^i^	88.50 (9)	N1—C11—H11	125.3
O4—Cu1—O3^i^	167.17 (7)	C3—C4—H4A	109.5
O2—Cu1—O1^i^	167.09 (7)	C3—C4—H4B	109.5
O4—Cu1—O1^i^	89.57 (9)	H4A—C4—H4B	109.5
O3^i^—Cu1—O1^i^	88.85 (9)	C3—C4—H4C	109.5
O2—Cu1—O5	103.44 (8)	H4A—C4—H4C	109.5
O4—Cu1—O5	90.86 (7)	H4B—C4—H4C	109.5
O3^i^—Cu1—O5	101.85 (7)	O6—C5—O5	121.9 (3)
O1^i^—Cu1—O5	89.47 (8)	O6—C5—C6	119.6 (2)
O2—Cu1—Cu1^i^	84.72 (5)	O5—C5—C6	118.5 (2)
O4—Cu1—Cu1^i^	80.15 (5)	C7—C8—H8A	109.5
O3^i^—Cu1—Cu1^i^	87.02 (5)	C7—C8—H8B	109.5
O1^i^—Cu1—Cu1^i^	82.53 (5)	H8A—C8—H8B	109.5
O5—Cu1—Cu1^i^	167.97 (6)	C7—C8—H8C	109.5
O7—Cu2—O9	89.95 (8)	H8A—C8—H8C	109.5
O7—Cu2—O10^ii^	89.75 (8)	H8B—C8—H8C	109.5
O9—Cu2—O10^ii^	167.61 (7)	C1—C2—H2A	109.5
O7—Cu2—O8^ii^	167.52 (7)	C1—C2—H2B	109.5
O9—Cu2—O8^ii^	88.09 (8)	H2A—C2—H2B	109.5
O10^ii^—Cu2—O8^ii^	89.52 (8)	C1—C2—H2C	109.5
O7—Cu2—O6	102.37 (8)	H2A—C2—H2C	109.5
O9—Cu2—O6	100.56 (7)	H2B—C2—H2C	109.5
O10^ii^—Cu2—O6	91.60 (7)	C12—C13—N1	106.4 (3)
O8^ii^—Cu2—O6	90.10 (7)	C12—C13—H13	126.8
O7—Cu2—Cu2^ii^	83.53 (5)	N1—C13—H13	126.8
O9—Cu2—Cu2^ii^	86.21 (5)	N1—C14—C15	111.6 (2)
O10^ii^—Cu2—Cu2^ii^	81.44 (5)	N1—C14—H14A	109.3
O8^ii^—Cu2—Cu2^ii^	84.05 (5)	C15—C14—H14A	109.3
O6—Cu2—Cu2^ii^	170.92 (5)	N1—C14—H14B	109.3
C7—O7—Cu2	124.17 (16)	C15—C14—H14B	109.3
C5—O5—Cu1	139.44 (18)	H14A—C14—H14B	108.0
C1—O2—Cu1	122.82 (16)	C14—C15—H15A	109.5
C7—O8—Cu2^ii^	122.70 (17)	C14—C15—H15B	109.5
C3—O4—Cu1	127.95 (15)	H15A—C15—H15B	109.5
C5—O6—Cu2	141.70 (19)	C14—C15—H15C	109.5
C9—O9—Cu2	120.40 (15)	H15A—C15—H15C	109.5
C9—O10—Cu2^ii^	126.01 (17)	H15B—C15—H15C	109.5
C1—O1—Cu1^i^	124.53 (17)	C13—C12—N2	108.1 (3)
C3—O3—Cu1^i^	119.18 (17)	C13—C12—H12	125.9
C11—N1—C13	108.5 (2)	N2—C12—H12	125.9
C11—N1—C14	126.6 (2)	C9—C10—H10A	109.5
C13—N1—C14	124.9 (2)	C9—C10—H10B	109.5
C11—N2—C12	107.7 (2)	H10A—C10—H10B	109.5
C11—N2—C16	126.7 (3)	C9—C10—H10C	109.5
C12—N2—C16	125.6 (3)	H10A—C10—H10C	109.5
O8—C7—O7	125.5 (2)	H10B—C10—H10C	109.5
O8—C7—C8	117.3 (2)	C5—C6—H6A	109.5
O7—C7—C8	117.2 (2)	C5—C6—H6B	109.5
O2—C1—O1	125.2 (2)	H6A—C6—H6B	109.5
O2—C1—C2	118.1 (2)	C5—C6—H6C	109.5
O1—C1—C2	116.7 (2)	H6A—C6—H6C	109.5
O10—C9—O9	125.9 (2)	H6B—C6—H6C	109.5
O10—C9—C10	116.6 (2)	N2—C16—H16A	109.5
O9—C9—C10	117.5 (2)	N2—C16—H16B	109.5
O4—C3—O3	125.6 (2)	H16A—C16—H16B	109.5
O4—C3—C4	116.9 (2)	N2—C16—H16C	109.5
O3—C3—C4	117.4 (2)	H16A—C16—H16C	109.5
N2—C11—N1	109.4 (2)	H16B—C16—H16C	109.5
			
Cu2^ii^—O8—C7—O7	0.4 (3)	C12—N2—C11—N1	0.3 (3)
Cu2^ii^—O8—C7—C8	−178.57 (17)	C16—N2—C11—N1	179.2 (2)
Cu2—O7—C7—O8	−2.0 (4)	C13—N1—C11—N2	0.0 (3)
Cu2—O7—C7—C8	176.93 (17)	C14—N1—C11—N2	177.5 (2)
Cu1—O2—C1—O1	−2.9 (4)	Cu2—O6—C5—O5	−174.30 (18)
Cu1—O2—C1—C2	177.00 (18)	Cu2—O6—C5—C6	5.5 (4)
Cu1^i^—O1—C1—O2	5.8 (4)	Cu1—O5—C5—O6	−167.95 (18)
Cu1^i^—O1—C1—C2	−174.07 (18)	Cu1—O5—C5—C6	12.2 (4)
Cu2^ii^—O10—C9—O9	−1.2 (4)	C11—N1—C13—C12	−0.2 (3)
Cu2^ii^—O10—C9—C10	179.0 (2)	C14—N1—C13—C12	−177.8 (3)
Cu2—O9—C9—O10	−0.4 (4)	C11—N1—C14—C15	−105.3 (3)
Cu2—O9—C9—C10	179.4 (2)	C13—N1—C14—C15	71.8 (4)
Cu1—O4—C3—O3	3.2 (4)	N1—C13—C12—N2	0.4 (4)
Cu1—O4—C3—C4	−176.27 (19)	C11—N2—C12—C13	−0.4 (3)
Cu1^i^—O3—C3—O4	−2.8 (4)	C16—N2—C12—C13	−179.3 (3)
Cu1^i^—O3—C3—C4	176.66 (19)		

Symmetry codes: (i) −*x*+2, −*y*+2, −*z*+2; (ii) −*x*+1, −*y*+2, −*z*+1.

### catena-Poly[1-ethyl-3-methylimidazolium [[tetra-µ-acetato-dicuprate(II)]-µ-acetato]] (3) Hydrogen-bond geometry (Å, º)

**Table:**

*D*—H···*A*	*D*—H	H···*A*	*D*···*A*	*D*—H···*A*
C6—H6*A*···O7	0.98	2.50	3.320 (4)	141
C14—H14*A*···O5^iii^	0.99	2.47	3.329 (3)	145
C13—H13···O8^iv^	0.95	2.38	3.229 (4)	148
C8—H8*C*···O7^v^	0.98	2.55	3.522 (4)	170
C11—H11···O1^i^	0.95	2.40	3.317 (3)	162
C11—H11···O5	0.95	2.55	3.192 (3)	125

Symmetry codes: (i) −*x*+2, −*y*+2, −*z*+2; (iii) *x*, *y*−1, *z*; (iv) −*x*+1, −*y*+1, −*z*+1; (v) −*x*+2, −*y*+2, −*z*+1.

### Bis(1-ethyl-3-methylimidazolium) tetra-µ-acetato-bis[aquacopper(II)] tetra-µ-acetato-bis[acetatocuprate(II)] dihydrate (4) Crystal data

**Table:**

(C_6_H_11_N_2_)_2_[Cu_2_(C_2_H_3_O_2_)_6_][Cu_2_(C_2_H_3_O_2_)_4_(H_2_O)_2_]·2H_2_O	*Z* = 1
*M**_r_* = 1139.00	*F*(000) = 588
Triclinic, *P*1	*D*_x_ = 1.615 Mg m^−^^3^
*a* = 7.9526 (5) Å	Mo *K*α radiation, λ = 0.71073 Å
*b* = 8.0951 (5) Å	Cell parameters from 4961 reflections
*c* = 18.8886 (11) Å	θ = 2.6–29.6°
α = 79.1770 (16)°	µ = 1.88 mm^−^^1^
β = 78.9500 (16)°	*T* = 198 K
γ = 89.9320 (15)°	Prism, blue
*V* = 1171.46 (12) Å^3^	0.30 × 0.27 × 0.22 mm

### Bis(1-ethyl-3-methylimidazolium) tetra-µ-acetato-bis[aquacopper(II)] tetra-µ-acetato-bis[acetatocuprate(II)] dihydrate (4) Data collection

**Table:**

Bruker Kappa APEX DUO CCD diffractometer	4775 independent reflections
Radiation source: fine-focus sealed tube	3593 reflections with *I* > 2σ(*I*)
Graphite monochromator	*R*_int_ = 0.037
φ and ω scans	θ_max_ = 26.4°, θ_min_ = 1.1°
Absorption correction: multi-scan (SADABS; Bruker, 2015)	*h* = −9→9
*T*_min_ = 0.605, *T*_max_ = 0.685	*k* = −10→10
20914 measured reflections	*l* = −23→23

### Bis(1-ethyl-3-methylimidazolium) tetra-µ-acetato-bis[aquacopper(II)] tetra-µ-acetato-bis[acetatocuprate(II)] dihydrate (4) Refinement

**Table:**

Refinement on *F*^2^	72 restraints
Least-squares matrix: full	Hydrogen site location: mixed
*R*[*F*^2^ > 2σ(*F*^2^)] = 0.034	H atoms treated by a mixture of independent and constrained refinement
*wR*(*F*^2^) = 0.101	*w* = 1/[σ^2^(*F*_o_^2^) + (0.038*P*)^2^] where *P* = (*F*_o_^2^ + 2*F*_c_^2^)/3
*S* = 1.42	(Δ/σ)_max_ = 0.001
4775 reflections	Δρ_max_ = 0.40 e Å^−^^3^
307 parameters	Δρ_min_ = −0.56 e Å^−^^3^

### Bis(1-ethyl-3-methylimidazolium) tetra-µ-acetato-bis[aquacopper(II)] tetra-µ-acetato-bis[acetatocuprate(II)] dihydrate (4) Special details

**Table:**

Geometry. All esds (except the esd in the dihedral angle between two l.s. planes) are estimated using the full covariance matrix. The cell esds are taken into account individually in the estimation of esds in distances, angles and torsion angles; correlations between esds in cell parameters are only used when they are defined by crystal symmetry. An approximate (isotropic) treatment of cell esds is used for estimating esds involving l.s. planes.

### Bis(1-ethyl-3-methylimidazolium) tetra-µ-acetato-bis[aquacopper(II)] tetra-µ-acetato-bis[acetatocuprate(II)] dihydrate (4) Fractional atomic coordinates and isotropic or equivalent isotropic displacement parameters (Å^2^)

**Table:**

	*x*	*y*	*z*	*U*_iso_*/*U*_eq_	
Cu1	0.91056 (4)	0.44336 (4)	0.06690 (2)	0.01957 (12)	
Cu2	0.09960 (5)	0.06109 (5)	0.43531 (2)	0.02361 (12)	
O1	0.7251 (3)	0.5696 (3)	0.02852 (12)	0.0273 (5)	
O2	0.8722 (3)	0.6597 (3)	−0.08616 (12)	0.0277 (5)	
O3	0.9897 (3)	0.6526 (3)	0.09078 (13)	0.0316 (6)	
O4	1.1418 (3)	0.7477 (3)	−0.02273 (13)	0.0289 (5)	
O11	0.4649 (3)	0.2016 (3)	0.23534 (13)	0.0355 (6)	
O6	0.2604 (3)	0.1127 (3)	0.49611 (13)	0.0356 (6)	
O5	0.7795 (3)	0.3431 (3)	0.17670 (13)	0.0354 (6)	
H5	0.8414	0.2734	0.1976	0.053*	
O9	−0.1846 (3)	0.1702 (3)	0.55415 (14)	0.0366 (6)	
O7	0.0942 (3)	0.0128 (3)	0.60447 (12)	0.0333 (6)	
O8	−0.0205 (3)	0.2707 (3)	0.44485 (14)	0.0386 (6)	
O10	0.2485 (3)	0.1352 (3)	0.32793 (13)	0.0378 (6)	
C1	0.7382 (4)	0.6483 (4)	−0.03698 (18)	0.0220 (7)	
N2	0.4809 (4)	0.7850 (4)	0.19273 (18)	0.0449 (8)	
C3	1.0864 (4)	0.7590 (4)	0.04339 (19)	0.0256 (7)	
N1	0.2327 (5)	0.6858 (4)	0.25088 (17)	0.0476 (9)	
C5	0.2322 (4)	0.0782 (4)	0.56465 (18)	0.0246 (7)	
O12	0.0083 (5)	0.1154 (5)	0.23115 (18)	0.0715 (10)	
C9	0.3888 (4)	0.2120 (4)	0.29812 (18)	0.0259 (7)	
C7	−0.1375 (4)	0.2826 (4)	0.4983 (2)	0.0317 (8)	
C2	0.5825 (4)	0.7351 (4)	−0.0578 (2)	0.0310 (8)	
H2A	0.6105	0.7943	−0.1090	0.046*	
H2B	0.5461	0.8161	−0.0258	0.046*	
H2C	0.4896	0.6514	−0.0520	0.046*	
C4	1.1405 (5)	0.9151 (4)	0.0676 (2)	0.0367 (9)	
H4A	1.2383	0.9718	0.0315	0.055*	
H4B	1.1735	0.8834	0.1156	0.055*	
H4C	1.0447	0.9913	0.0713	0.055*	
C6	0.3746 (5)	0.1154 (5)	0.6017 (2)	0.0392 (9)	
H6A	0.4806	0.0680	0.5794	0.059*	
H6B	0.3451	0.0651	0.6541	0.059*	
H6C	0.3911	0.2375	0.5960	0.059*	
C10	0.4686 (5)	0.3206 (5)	0.3413 (2)	0.0465 (10)	
H10A	0.5846	0.3585	0.3149	0.070*	
H10B	0.4743	0.2547	0.3899	0.070*	
H10C	0.3985	0.4186	0.3471	0.070*	
C12	0.4448 (6)	0.6441 (5)	0.1662 (2)	0.0482 (10)	
H12	0.5179	0.5991	0.1290	0.058*	
C13	0.2902 (6)	0.5817 (5)	0.2016 (2)	0.0490 (10)	
H13	0.2315	0.4858	0.1944	0.059*	
C11	0.3486 (5)	0.8073 (5)	0.2436 (2)	0.0471 (10)	
H11	0.3385	0.8960	0.2705	0.056*	
C8	−0.2333 (5)	0.4447 (5)	0.4944 (2)	0.0477 (10)	
H8A	−0.3362	0.4323	0.4742	0.072*	
H8B	−0.1591	0.5367	0.4627	0.072*	
H8C	−0.2665	0.4698	0.5438	0.072*	
C16	0.6413 (5)	0.8940 (6)	0.1651 (3)	0.0675 (14)	
H16A	0.6340	0.9661	0.1180	0.101*	
H16B	0.7406	0.8228	0.1584	0.101*	
H16C	0.6539	0.9644	0.2008	0.101*	
C14	0.0722 (6)	0.6606 (8)	0.3061 (3)	0.0893 (19)	
H14A	0.0901	0.5741	0.3483	0.107*	
H14B	0.0479	0.7670	0.3242	0.107*	
C15	−0.0685 (7)	0.6127 (11)	0.2825 (3)	0.140 (4)	
H15A	−0.0972	0.7036	0.2451	0.209*	
H15B	−0.1654	0.5886	0.3242	0.209*	
H15C	−0.0442	0.5115	0.2615	0.209*	
H5B	0.678 (3)	0.307 (5)	0.202 (2)	0.062 (14)*	
H1O	−0.052 (5)	0.106 (6)	0.2779 (14)	0.074*	
H2O	0.090 (4)	0.146 (6)	0.251 (2)	0.074*	

### Bis(1-ethyl-3-methylimidazolium) tetra-µ-acetato-bis[aquacopper(II)] tetra-µ-acetato-bis[acetatocuprate(II)] dihydrate (4) Atomic displacement parameters (Å^2^)

**Table:**

	*U*^11^	*U*^22^	*U*^33^	*U*^12^	*U*^13^	*U*^23^
Cu1	0.0164 (2)	0.0196 (2)	0.0217 (2)	−0.00175 (15)	−0.00105 (16)	−0.00408 (16)
Cu2	0.0196 (2)	0.0277 (2)	0.0207 (2)	−0.00132 (16)	0.00128 (16)	−0.00271 (17)
O1	0.0197 (12)	0.0294 (12)	0.0302 (13)	0.0025 (9)	−0.0013 (10)	−0.0033 (10)
O2	0.0187 (12)	0.0347 (13)	0.0275 (13)	0.0010 (10)	−0.0030 (10)	−0.0025 (10)
O3	0.0368 (14)	0.0260 (12)	0.0329 (14)	−0.0069 (10)	−0.0032 (11)	−0.0115 (10)
O4	0.0296 (13)	0.0234 (12)	0.0345 (14)	−0.0046 (10)	−0.0049 (11)	−0.0088 (10)
O11	0.0304 (13)	0.0434 (15)	0.0287 (14)	−0.0085 (11)	0.0083 (11)	−0.0109 (11)
O6	0.0279 (13)	0.0488 (16)	0.0276 (14)	−0.0085 (11)	−0.0039 (11)	−0.0026 (11)
O5	0.0292 (14)	0.0439 (16)	0.0265 (14)	−0.0061 (12)	0.0031 (11)	0.0011 (11)
O9	0.0312 (14)	0.0347 (14)	0.0415 (16)	0.0082 (11)	0.0008 (12)	−0.0087 (12)
O7	0.0253 (13)	0.0492 (16)	0.0243 (13)	−0.0052 (11)	−0.0029 (10)	−0.0063 (11)
O8	0.0412 (15)	0.0295 (14)	0.0399 (16)	0.0062 (11)	0.0000 (12)	−0.0020 (11)
O10	0.0242 (13)	0.0605 (17)	0.0232 (13)	−0.0128 (12)	0.0036 (10)	−0.0027 (12)
C1	0.0207 (16)	0.0183 (16)	0.0291 (18)	−0.0031 (12)	−0.0061 (14)	−0.0085 (13)
N2	0.045 (2)	0.049 (2)	0.046 (2)	0.0203 (16)	−0.0117 (16)	−0.0194 (17)
C3	0.0210 (17)	0.0214 (17)	0.039 (2)	0.0035 (13)	−0.0130 (15)	−0.0093 (15)
N1	0.056 (2)	0.053 (2)	0.035 (2)	0.0090 (17)	0.0004 (16)	−0.0206 (16)
C5	0.0262 (18)	0.0208 (16)	0.0271 (19)	0.0037 (13)	−0.0051 (14)	−0.0056 (13)
O12	0.087 (3)	0.080 (2)	0.057 (2)	0.012 (2)	−0.0423 (19)	−0.0094 (19)
C9	0.0269 (18)	0.0246 (17)	0.0231 (18)	0.0011 (14)	−0.0010 (15)	−0.0006 (14)
C7	0.0274 (19)	0.0299 (19)	0.040 (2)	0.0013 (15)	−0.0101 (16)	−0.0091 (16)
C2	0.0200 (17)	0.0293 (18)	0.043 (2)	0.0009 (14)	−0.0094 (15)	−0.0026 (16)
C4	0.040 (2)	0.0249 (18)	0.051 (2)	−0.0036 (16)	−0.0151 (19)	−0.0178 (17)
C6	0.030 (2)	0.050 (2)	0.041 (2)	0.0032 (17)	−0.0139 (17)	−0.0107 (18)
C10	0.057 (3)	0.045 (2)	0.033 (2)	−0.021 (2)	0.0052 (19)	−0.0097 (18)
C12	0.060 (3)	0.047 (2)	0.044 (3)	0.027 (2)	−0.013 (2)	−0.022 (2)
C13	0.067 (3)	0.043 (2)	0.041 (2)	0.016 (2)	−0.009 (2)	−0.0196 (19)
C11	0.049 (2)	0.051 (3)	0.048 (3)	0.0108 (19)	−0.012 (2)	−0.025 (2)
C8	0.048 (3)	0.034 (2)	0.062 (3)	0.0153 (18)	−0.009 (2)	−0.0132 (19)
C16	0.040 (3)	0.059 (3)	0.110 (4)	0.009 (2)	−0.012 (3)	−0.032 (3)
C14	0.067 (3)	0.121 (5)	0.079 (4)	−0.023 (3)	0.024 (3)	−0.055 (4)
C15	0.068 (4)	0.289 (11)	0.072 (4)	−0.041 (5)	0.008 (3)	−0.082 (6)

### Bis(1-ethyl-3-methylimidazolium) tetra-µ-acetato-bis[aquacopper(II)] tetra-µ-acetato-bis[acetatocuprate(II)] dihydrate (4) Geometric parameters (Å, º)

**Table:**

Cu1—O3	1.967 (2)	N1—C14	1.473 (5)
Cu1—O1	1.968 (2)	C5—C6	1.499 (4)
Cu1—O4^i^	1.970 (2)	O12—H1O	0.910 (18)
Cu1—O2^i^	1.984 (2)	O12—H2O	0.874 (19)
Cu1—O5	2.142 (2)	C9—C10	1.520 (5)
Cu1—Cu1^i^	2.6469 (7)	C7—C8	1.513 (5)
Cu2—O8	1.967 (2)	C2—H2A	0.9800
Cu2—O6	1.968 (2)	C2—H2B	0.9800
Cu2—O9^ii^	1.978 (2)	C2—H2C	0.9800
Cu2—O7^ii^	1.978 (2)	C4—H4A	0.9800
Cu2—O10	2.121 (2)	C4—H4B	0.9800
Cu2—Cu2^ii^	2.6592 (8)	C4—H4C	0.9800
O1—C1	1.266 (4)	C6—H6A	0.9800
O2—C1	1.263 (4)	C6—H6B	0.9800
O2—Cu1^i^	1.984 (2)	C6—H6C	0.9800
O3—C3	1.258 (4)	C10—H10A	0.9800
O4—C3	1.263 (4)	C10—H10B	0.9800
O4—Cu1^i^	1.970 (2)	C10—H10C	0.9800
O11—C9	1.242 (4)	C12—C13	1.331 (6)
O6—C5	1.248 (4)	C12—H12	0.9500
O5—H5	0.8400	C13—H13	0.9500
O5—H5B	0.878 (18)	C11—H11	0.9500
O9—C7	1.253 (4)	C8—H8A	0.9800
O9—Cu2^ii^	1.978 (2)	C8—H8B	0.9800
O7—C5	1.259 (4)	C8—H8C	0.9800
O7—Cu2^ii^	1.978 (2)	C16—H16A	0.9800
O8—C7	1.253 (4)	C16—H16B	0.9800
O10—C9	1.256 (4)	C16—H16C	0.9800
C1—C2	1.504 (4)	C14—C15	1.363 (6)
N2—C11	1.319 (5)	C14—H14A	0.9900
N2—C12	1.381 (5)	C14—H14B	0.9900
N2—C16	1.501 (5)	C15—H15A	0.9800
C3—C4	1.511 (4)	C15—H15B	0.9800
N1—C11	1.318 (5)	C15—H15C	0.9800
N1—C13	1.385 (5)		
			
O3—Cu1—O1	88.30 (10)	O8—C7—O9	125.6 (3)
O3—Cu1—O4^i^	168.28 (10)	O8—C7—C8	117.4 (3)
O1—Cu1—O4^i^	90.23 (9)	O9—C7—C8	117.0 (3)
O3—Cu1—O2^i^	88.82 (10)	C1—C2—H2A	109.5
O1—Cu1—O2^i^	168.07 (9)	C1—C2—H2B	109.5
O4^i^—Cu1—O2^i^	90.24 (9)	H2A—C2—H2B	109.5
O3—Cu1—O5	94.73 (10)	C1—C2—H2C	109.5
O1—Cu1—O5	99.95 (9)	H2A—C2—H2C	109.5
O4^i^—Cu1—O5	96.98 (10)	H2B—C2—H2C	109.5
O2^i^—Cu1—O5	91.83 (9)	C3—C4—H4A	109.5
O3—Cu1—Cu1^i^	85.55 (7)	C3—C4—H4B	109.5
O1—Cu1—Cu1^i^	83.63 (7)	H4A—C4—H4B	109.5
O4^i^—Cu1—Cu1^i^	82.74 (7)	C3—C4—H4C	109.5
O2^i^—Cu1—Cu1^i^	84.60 (7)	H4A—C4—H4C	109.5
O5—Cu1—Cu1^i^	176.41 (7)	H4B—C4—H4C	109.5
O8—Cu2—O6	91.32 (11)	C5—C6—H6A	109.5
O8—Cu2—O9^ii^	167.41 (10)	C5—C6—H6B	109.5
O6—Cu2—O9^ii^	88.28 (10)	H6A—C6—H6B	109.5
O8—Cu2—O7^ii^	87.89 (10)	C5—C6—H6C	109.5
O6—Cu2—O7^ii^	167.25 (10)	H6A—C6—H6C	109.5
O9^ii^—Cu2—O7^ii^	89.73 (10)	H6B—C6—H6C	109.5
O8—Cu2—O10	99.52 (10)	C9—C10—H10A	109.5
O6—Cu2—O10	101.45 (9)	C9—C10—H10B	109.5
O9^ii^—Cu2—O10	92.89 (10)	H10A—C10—H10B	109.5
O7^ii^—Cu2—O10	91.22 (9)	C9—C10—H10C	109.5
O8—Cu2—Cu2^ii^	84.34 (7)	H10A—C10—H10C	109.5
O6—Cu2—Cu2^ii^	83.49 (7)	H10B—C10—H10C	109.5
O9^ii^—Cu2—Cu2^ii^	83.11 (7)	C13—C12—N2	108.5 (4)
O7^ii^—Cu2—Cu2^ii^	83.77 (7)	C13—C12—H12	125.8
O10—Cu2—Cu2^ii^	173.59 (7)	N2—C12—H12	125.8
C1—O1—Cu1	124.3 (2)	C12—C13—N1	105.6 (4)
C1—O2—Cu1^i^	122.4 (2)	C12—C13—H13	127.2
C3—O3—Cu1	121.4 (2)	N1—C13—H13	127.2
C3—O4—Cu1^i^	124.4 (2)	N1—C11—N2	108.6 (4)
C5—O6—Cu2	124.5 (2)	N1—C11—H11	125.7
Cu1—O5—H5	109.5	N2—C11—H11	125.7
Cu1—O5—H5B	142 (3)	C7—C8—H8A	109.5
H5—O5—H5B	100.1	C7—C8—H8B	109.5
C7—O9—Cu2^ii^	123.8 (2)	H8A—C8—H8B	109.5
C5—O7—Cu2^ii^	123.4 (2)	C7—C8—H8C	109.5
C7—O8—Cu2	122.9 (2)	H8A—C8—H8C	109.5
C9—O10—Cu2	137.9 (2)	H8B—C8—H8C	109.5
O2—C1—O1	125.0 (3)	N2—C16—H16A	109.5
O2—C1—C2	117.5 (3)	N2—C16—H16B	109.5
O1—C1—C2	117.5 (3)	H16A—C16—H16B	109.5
C11—N2—C12	107.8 (4)	N2—C16—H16C	109.5
C11—N2—C16	127.5 (4)	H16A—C16—H16C	109.5
C12—N2—C16	124.7 (4)	H16B—C16—H16C	109.5
O3—C3—O4	125.9 (3)	C15—C14—N1	115.7 (5)
O3—C3—C4	117.1 (3)	C15—C14—H14A	108.3
O4—C3—C4	117.0 (3)	N1—C14—H14A	108.3
C11—N1—C13	109.4 (4)	C15—C14—H14B	108.3
C11—N1—C14	124.7 (4)	N1—C14—H14B	108.3
C13—N1—C14	125.7 (4)	H14A—C14—H14B	107.4
O6—C5—O7	124.8 (3)	C14—C15—H15A	109.5
O6—C5—C6	117.2 (3)	C14—C15—H15B	109.5
O7—C5—C6	118.0 (3)	H15A—C15—H15B	109.5
H1O—O12—H2O	82 (3)	C14—C15—H15C	109.5
O11—C9—O10	122.7 (3)	H15A—C15—H15C	109.5
O11—C9—C10	119.0 (3)	H15B—C15—H15C	109.5
O10—C9—C10	118.3 (3)		

Symmetry codes: (i) −*x*+2, −*y*+1, −*z*; (ii) −*x*, −*y*, −*z*+1.

### Bis(1-ethyl-3-methylimidazolium) tetra-µ-acetato-bis[aquacopper(II)] tetra-µ-acetato-bis[acetatocuprate(II)] dihydrate (4) Hydrogen-bond geometry (Å, º)

**Table:**

*D*—H···*A*	*D*—H	H···*A*	*D*···*A*	*D*—H···*A*
O12—H2*O*···O10	0.84 (2)	2.14 (3)	2.912 (4)	152 (4)
O5—H5*B*···O11	0.85 (2)	1.86 (2)	2.695 (3)	171 (4)
C14—H14*B*···O7^iii^	0.99	2.57	3.530 (6)	162
C16—H16*C*···O11^iv^	0.98	2.56	3.239 (5)	126
C16—H16*B*···O3	0.98	2.65	3.598 (5)	162
C11—H11···O10^iv^	0.95	2.44	3.365 (4)	166
C11—H11···O11^iv^	0.95	2.59	3.291 (4)	131
C12—H12···O1	0.95	2.30	3.224 (4)	163
C10—H10*B*···O6	0.98	2.47	3.241 (4)	136
C2—H2*C*···O1^v^	0.98	2.40	3.371 (3)	173
C2—H2*A*···O11^v^	0.98	2.58	3.387 (4)	140
O5—H5···O12^vi^	0.84	1.96	2.789 (4)	170

Symmetry codes: (iii) −*x*, −*y*+1, −*z*+1; (iv) *x*, *y*+1, *z*; (v) −*x*+1, −*y*+1, −*z*; (vi) *x*+1, *y*, *z*.
